# Fluoride-Free MXene–Polymer Composites for Li-Metal and Li–S Batteries: Comparative Synthesis Methods, Integration Rules, Challenges, and Future Directions

**DOI:** 10.3390/polym17233109

**Published:** 2025-11-23

**Authors:** Truong Le Khang, Joonho Bae

**Affiliations:** Department of Physics and Semiconductor Science, Gachon University, Seongnam-si 13120, Republic of Korea; khangle123@gachon.ac.kr

**Keywords:** MXene–polymer composites, fluoride-free MXene, lithium metal batteries, lithium–sulfur batteries

## Abstract

MXene–polymer hybrids combine the high in-plane conductivity and rich surface chemistry of MXenes with the processability and mechanical tunability of polymers for lithium-metal and lithium–sulfur batteries. Most reported systems still rely on HF-etched MXenes, introducing F-rich terminations, safety and waste issues, and poorly controlled surfaces. This review instead centers on fluoride-free synthesis routes, benchmarks them against HF methods, and translates route–termination relationships into practical rules for choosing polymer backbones. We track the evolution from early linear hosts such as PEO- and PVDF-type polymers to polar nitrile or carbonyl matrices, crosslinked and ionogel networks, and emerging biopolymers and COF-type porous frameworks that are co-designed with MXene terminations to regulate ion transport, interfacial chemistry, and mechanical robustness. These chemistry–backbone pairings are linked to five scalable fabrication modes, including solution blending and film casting, in situ polymerization, surface grafting, layer-by-layer assembly, and electrospinning, and to roles as solid or quasi-solid electrolytes, artificial interphases, separator-like coatings, and electrode-facing architectures. Finally, we highlight key evidence gaps and reporting standards needed to de-risk scale-up of green MXene–polymer batteries.

## 1. Introduction

Electrochemical energy storage supports the electrification of transport and the integration of variable renewables into power systems. Lithium-ion technology set the modern benchmark for cycle life and efficiency, yet limits in safety, cost at pack level, and practical energy density motivate chemistries that move beyond intercalation toward lithium-metal anodes and conversion-type cathodes [1,2,3,4]. In this review, lithium batteries refer to lithium-metal-based chemistries, namely lithium-metal batteries and lithium–sulfur cells. Both target step-change specific energy yet are limited by interfacial failure modes. Lithium metal requires controlled plating and stripping to avoid dendritic growth and unstable solid–electrolyte interphases. Lithium–sulfur couples an electronically insulating sulfur cathode with soluble polysulfide intermediates and large conversion strains that drive parasitic reactions [5,6,7]. These constraints shift the focus from simply increasing active mass to engineering transport, interphase chemistry, and mechanical integrity through electrolytes, artificial interphases, and separator-like layers. MXenes are two-dimensional transition-metal carbides and nitrides with high in-plane conductivity, tunable surface terminations, lamellar galleries, and polar surfaces. When combined with polymers, they form electronically continuous yet chemically addressable scaffolds that can be processed into membranes, binders, and interlayers [8,9,10]. In lithium-metal batteries, MXene–polymer architectures can homogenize interfacial currents at the anode and strengthen and stabilize the SEI. They also reinforce high-loading cathodes by supplying continuous electron pathways and compliant ion conduction frameworks. In lithium–sulfur cells, polar MXene surfaces adsorb and catalyze polysulfide conversion, while the polymer matrix preserves wetting, ion transport, and dimensional stability. Together these functions enable permselective interlayers and safer polymer-rich electrolytes [2,3,4,5,6,11]. Because these effects depend strongly on surface chemistry, the synthesis route becomes decisive. The resulting terminations control dispersion in polymer media, polymer affinity, and the reactions at working interfaces. Most MXenes to date have been prepared by HF or in situ HF routes. These methods are efficient and scalable but entail stringent safety and waste management and commonly yield fluorine-rich surfaces that complicate hydrophilicity, composite uniformity, and durable interphase formation [6,10,12]. Greener strategies have therefore emerged to reduce hazards and steer the termination palette toward O/OH or programmed halides that integrate more predictably with polymers. Representative approaches include Lewis-acid molten-salt etching that affords Cl-terminated MXenes with tunable work function and spacing, alkali or hydrothermal treatments that avoid fluoride, halogen and iodine-assisted routes in anhydrous media, and electrochemical de-aluminization in mixed LiOH and LiCl that widens galleries. These chemistries improve environmental, health, and safety profiles and produce surfaces that align with polymer processing and more benign interphase formation. Despite this momentum, there are still few studies that explicitly pair fluoride-free MXenes with polymers in working lithium-metal and lithium–sulfur cells. As a result, key questions remain about termination control, residue management, processing windows, and reproducibility. This review addresses that gap in four steps. First, we compare HF-based and green syntheses and map their characteristic terminations. Second, we explain how MXene surface chemistry underpins polymer compatibility. Third, we summarize composite-forming methods and design rules that link flake loading, lateral size, and in-plane alignment to ionic conduction and electronic insulation. Finally, we survey applications from both routes across lithium-metal and lithium–sulfur batteries and highlight cross-cutting challenges for deployment, including corrosion and hydrogen suppression, halide or oxidant cleanup, thermal and field uniformity, and scale-up reproducibility.

## 2. MXenes: Structure, Synthesis, Function

MXenes are a large family of two-dimensional transition-metal carbides and nitrides with the general formula M_n+1_X_n_T_x_ (M = early transition metal; X = C and/or N. T_x_ = surface terminations such as -O, -OH, -F, -Cl). They are typically produced by selectively removing the A element from layered MAX phases, resulting in conductive lamellar sheets whose interlayer spacing and surface chemistry are highly tunable [8]. Beyond the “classic” monometallic carbides such as Ti_3_C_2_T_x_, Ti_2_CT_x_, Nb_2_CT_x_, and V_2_CT_x_, the family also includes nitrides and carbonitrides as well as ordered double MXenes such as Mo_2_TiC_2_T_x_. Across these chemistries, MXenes combine high in-plane electrical conductivity, abundant surface terminations, good colloidal dispersibility, and low-loading percolation, making them attractive building blocks for energy storage polymer composites [8,13]. Within this landscape, titanium-based MXenes, in particular Ti_3_C_2_T_x_, dominate energy-related studies and function as the de facto choice for polymer composites. The reasons are both practical and mechanistic. There is reliable access to Ti-MAX precursors and to etching routes that scale. Ti-based MXenes also offer robust flake quality with controllable terminations (typically -O, -OH, -F, and -Cl depending on synthesis), which enables predictable interfacial interactions. In addition, they provide a favorable balance of conductivity, hydrophilicity, and chemical stability in common processing media. In polymer electrolytes and in electrode binders, Ti-based MXenes can promote salt dissociation and ion transport through Lewis-acid–base and hydrogen bonding with ether, nitrile, or ionic backbones. They can reduce polymer crystallinity and thereby unlock room-temperature conductivity. They can also reinforce the mechanical modulus and raise the critical current density. In addition, they can provide continuous electron or heat pathways when desired. These attributes explain why Ti_3_C_2_T_x_–polymer systems recur across solid-state lithium and lithium–sulfur batteries and motivate the focus on how synthesis-controlled terminations govern polymer compatibility and device-level performance [13]. Regarding synthesis, the historical baseline is HF-based etching, including variants that generate HF in situ with LiF + HCl. This route efficiently removes the A layer and yields F-containing surfaces, but it also introduces well-known safety and waste management liabilities and can imprint termination variability that propagates into composite processing [14,15]. In parallel, fluorine-free or green strategies have emerged to minimize hazardous inputs, improve EHS profiles, and steer T_x_ toward chemistries better aligned with targeted polymers. Representative approaches include electrochemical routes in chloride media or ionic liquids, Lewis-acid molten-salt routes, and hydrothermal or alkaline routes [16].

### 2.1. HF-Based Synthesis of MXenes

HF etching remains the baseline method for producing delaminated MXenes. In the classic direct HF route (20–50 wt% HF at room temperature), first demonstrated by Naguib et al., the Al layer of Ti_3_AlC_2_ is removed to yield Ti_3_C_2_T_x_ sheets [10]. This process is simple and fast but introduces mixed F/OH/O terminations and demands stringent safety measures. In practice, the resulting F-terminated MXene surfaces have reduced polarity and interact weakly with many polar polymers, so HF-etched MXenes often require additional treatments or compatibilizers to form uniform composites [17,18].

Variants of HF etching (in situ HF from LiF + HCl or buffered HF via bifluoride salts) similarly exfoliate MXenes and expand the interlayer galleries [19,20,21]. For example, the LiF/HCl “in situ HF” method (Ghidiu et al.) produces large, clay-like Ti_3_C_2_T_x_ flakes, but it remains fluorine-bearing and tends to yield F-rich terminations that lower surface polarity [17,19,22]. Buffered HF salts (NH_4_HF_2_, KHF_2_, etc.) moderate the etch chemistry and enlarge c-spacing, but they too produce predominantly -F, -OH, -O surfaces and remain fluorine-bearing, requiring rigorous effluent management [20,23,24].

In summary, all HF-based etches efficiently de-aluminate the MAX phase to give conductive Ti_3_C_2_T_x_ nanosheets. However, they carry major drawbacks: persistent F-rich terminations (which lower polarity and hinder dispersion in polar polymers) and hazardous fluoride wastes that complicate scale-up and environmental management.

In practice, these limitations mean that HF-etched MXenes tend to form LiF-dominated, electronically insulating interphases on lithium metal. Thus, although HF etching gives rapid, high-yield access to delaminable Ti_3_C_2_T_x_, its F-terminated surfaces and toxic byproducts impose strict safety requirements and often necessitate post-etch modifications (e.g., extensive washing or chemical exchange) before polymer composite fabrication.

### 2.2. Green Synthesis of MXenes

Since HF-based routes are hazardous to operators and generate fluoride-bearing effluents, sustained research has focused on fluoride-free “green” syntheses to reduce toxicity and environmental burden while preserving etch selectivity and scalability. Shifting surface terminations away from F-rich toward O/OH or, in some cases, Cl provides interfacial benefits for energy storage, with O/OH terminations enhancing hydrophilicity, ion access, and Cl terminations from Lewis-acid media [25,26,27,28].

#### 2.2.1. Hydrothermal Etching Methods

Hydrothermal etching replaces HF or LiF/HCl with hot compressed water and dissolved reagents. Then, the A layer is removed while O and OH terminations form. Selective leaching proceeds through complexation or controlled redox under elevated temperature and autogenous pressure, yielding fluoride-free MXenes that delaminate cleanly with polymers and gel electrolytes. These oxygenated surfaces improve wettability and interfacial adhesion. Hence, inks, films, and binder-lean electrodes can be fabricated in water while avoiding persistent fluorine that often destabilizes polymer and electrolyte interphases.

##### Alkali Etching Method

Alkali hydrothermal etching replaces fluoride pathways by leaning on Bayer-type chemistry, where the concentrated base at elevated temperature converts Al in Ti_3_AlC_2_ into soluble [Al(OH)_4_]^−^ and opens Ti_3_C_2_ layers with oxygenated terminations. A strong HF-free benchmark was set by Li et al. in 2018, who used about 27.5 M NaOH near 270 °C to obtain multilayer Ti_3_C_2_T_x_ of roughly 92 wt% purity, with vacuum-filtered films near 314 F g^−1^ in 1 M H_2_SO_4_ [25]. The chemistry is anchored by aluminum leaching to aluminate with hydrogen evolution, after which a compact solid–solution step yields an O or OH terminated MXene:

(1) 2 Al + 2 NaOH + 2 H_2_O → 2 NaAlO_2_ + 3 H_2_

(2) 2 Al + 2 NaOH + 6 H_2_O → 2 NaAl(OH)_4_ + 3 H_2_

(3) Ti_3_AlC_2_ + NaOH + H_2_O → Ti_3_C_2_T_x_ (Tx=O/OH) + NaAl(OH)_4_ + H_2_

Within this NaOH window, O/OH-terminated Ti_3_C_2_T_x_ wets the aqueous binders and gel polymer electrolytes well, enabling uniform casting and low interfacial resistance. Product integrity depends on controlling base concentration, temperature, and time: under-etching leaves residual MAX, whereas overly harsh or long treatments promote alkali titanates. Hydrogen evolution and oxidation risks can be managed with corrosion-resistant autoclaves, limited free water, short dwells with rapid quenching, and thorough washing [16,25,27].

Using the Li et al. [25] benchmark as a guide, Khan et al. [29] defined a practical NaOH hydrothermal window near 280 °C for 15 h. By screening 22.5–40 M, they identified 30 M as the sweet spot for fluorine-free Ti_3_C_2_T_x_ with enlarged interlayer spacing and strong capacitance, and they emphasized the need to remove residual Al and Na–Ti–O during post-treatment. Yoon et al. [30] then refined the NaOH route by adding a brief acid rinse and rapid TMAOH delamination to deliver halogen-free monolayers with superior oxidation resistance to HF-derived analogs. In parallel, Kulkarni et al. [31] showed that KOH can yield multilayer Ti_3_C_2_–OH at gentler 180 °C in sealed PTFE vessels, though longer times or thermal pre-treatments readily divert the chemistry to potassium titanate, narrowing the viable window and helping explain why the KOH literature remains sparse. In practice, NaOH remains the most forgiving path from high-purity multilayers to monolayers. KOH offers a lower-temperature alternative but demands stricter control of reaction conditions. Both routes scale in autoclaves and provide fluorine-free surfaces for polymer–electrolyte integration, yet they still require rigorous washing, cation removal, and oxidation management before use.

##### Acid Etching Method

A complementary route relies on chloride complexation of the A element under hydrothermal conditions which lowers the barrier for extraction and allows oxygenated surfaces to emerge from bound water. Wang et al. in 2021 converted Mo_2_Ga_2_C to fluoride-free Mo_2_CT_x_ that carries Cl and O terminations using only HCl in an autoclave [32]. Building on this concept, Khan et al. recently showed that concentrated HCl hydrothermal activation of Ti_3_AlC_2_ (12 M HCl, 160–180 °C, several days) yields fluorine-free Ti_3_C_2_T_x_ with mixed OH/Cl terminations and expanded interlayer spacing. The optimized Ti_3_C_2_T_x_-6D-160 °C sample delivered a gravimetric capacitance of about 450 C g^−1^ at 5 mV s^−1^ in 1 M H_2_SO_4_, confirming that HCl-only etching can simultaneously remove A layers and engineer chloride-/oxide-rich surfaces for electrochemical applications [33].

A concise coupling for chloride–susceptible MAX systems reads as follows:

M_3_AC_2_ + 3 HCl + x H_2_O → M_3_C_2_(O/H)_x_ + ACl_3_ + (3/2) H_2_

Partial chloride can then be retained or exchanged during work-up to tune surface energy and adhesion in polar or fluorinated binders. The absence of persistent fluorine favors cleaner interphases in electrolytes. Attention to materials’ compatibility is essential since chloride can corrode autoclaves. A-species may reprecipitate on cool-down, and chloride residues must be removed thoroughly before composite fabrication [27,32].

##### Salt-Assisted Etching

Adding an oxidizing chloride salt to the alkali hydrothermal window lets the A layer leach in hot compressed water while the salt gently modulates redox and extraction. The earliest peer-reviewed example uses concentrated NaOH with a small dose of NaClO under hydrothermal conditions to convert Ti_3_AlC_2_ into fluorine-free, O/OH-terminated Ti_3_C_2_T_x_ bearing a thin in situ TiO_2_ skin that limits restacking. The product disperses in water and forms coherent films that deliver about 321 F g^−1^ with roughly 86% retention after 10,000 cycles in 1 M H_2_SO_4_, and no fluoride reagents or F-terminations are introduced [34]. This co-mediated window builds directly on the alkali-only route by Li and co-workers and remains compatible with polymer binders and gel electrolytes because the surfaces are wettable and fluorine-free. The salt provides a practical knob to tune rate and microstructure at modest temperatures [16,25]. The mechanistic picture can be captured as the following reactions.

Ti_3_AlC_2_ + NaOH + H_2_O + OCl^−^ → Ti_3_C_2_T_x_ (T_x_=O/OH) + NaAl(OH)_4_ + Cl^−^ + TiO_2_ + H_2_

A strong base removes Al as aluminate, hypochlorite accelerates kinetics, and a thin TiO_2_ layer spaces sheets to stabilize films. However, excess NaClO or long holds can over-oxidize the carbide core, so chloride must be rinsed thoroughly and hydrogen handled with suitable autoclaves and venting. With oxygen exclusion, short holds, rapid quenching, and rigorous washing, the salt-assisted route offers faster, fluoride-free processing and clean polymer–electrolyte interfaces.

##### Microwave–Hydrothermal Method

Microwave-driven etching is reshaping the MAX-to-MXene conversion, and it scales more cleanly than the classical alkaline hydrothermal. By coupling volumetric heating with rapid ion transport, reactions that once needed long soaks now finish faster and at lower temperatures, which lifts throughput and trims energy per gram. In this context, an HF-free open-vessel NaOH route uses modest microwave power and converts Ti_3_AlC_2_ to F-free Ti_3_C_2_T_x_ within minutes, which suits catalysis and polymer–electrolyte interfaces, though careful washing is still required to manage Na and OH terminations and to limit oxidation [35]. Building on the same logic yet adding tighter process control, a sealed microwave-assisted hydrothermal route reaches complete etching in tens of minutes around 180 °C, allows alkali strength to be tuned to drive efficient Al removal, and scales naturally in PTFE-lined vessels with good batch-to-batch reproducibility [36]. Relative to non-microwave–hydrothermal baselines, these HF-free microwave routes shorten the cycle from many hours to minutes or well under an hour, lower set points toward the 160–180 °C window, and promote more uniform interlayer activation that eases delamination and improves downstream mixing with polymers [35,36]. At pilot scale, the kinetic gains translate to higher daily output and lower energy intensity, although successful scale-up depends on electromagnetic field uniformity, robust stirring, and the use of appropriate multimode reactors. For a balanced conclusion, position the HF-free open-vessel route as the green and polymer-friendly choice for small to mid-scale work, and treat the sealed microwave–hydrothermal route as a tunable and straightforward path to parallelized scale-up in PTFE-lined autoclaves.

In summary, alkali and HCl-only routes provide fluorine-free Ti_3_C_2_T_x_ with O/OH or mixed Cl/O terminations that already support water-based casting, lamination, gel infiltration, and device-level performance. Salt-assisted and microwave–hydrothermal variants mainly tune ionic strength and kinetics to accelerate etching and stabilize flake quality while keeping surfaces fluorine-free.

#### 2.2.2. Electrochemical Etching Methods

Electrochemical etching offers a fluoride-free pathway in which chloride-assisted anodic de-aluminization, alkaline surface functionalization, and in situ intercalation together tune kinetics, surface terminations, and delamination. Sun et al. (2017) established feasibility in a three-electrode HCl system (1–2 M, 0.4–0.6 V vs. Ag/AgCl), converting Ti_2_AlC to Ti_2_CT_x_ with F-free O/OH (Cl) terminations while preserving the Ti–C backbone. However, extended polarization over-etches the outer MXene into a carbide-derived carbon skin, producing a MAX|MXene|CDC stratification that affects uniformity and conductivity [37]. The reported half-reactions are as follows:

(WE) Ti_2_AlC + yCl^−^ + (2x + z)H_2_O → Ti_2_C(OH)_2_xCl_γ_O_z + Al^3+^ + (x + z)H_2_ + (y + 3)e^−^

(CE) Al^3+^ + 3e^−^ → Al

Yang et al. (2018) removed this surface-limited bottleneck by using NH_4_Cl as the etchant and NH_4_OH as both the intercalant and base in a two-electrode cell. Under these conditions, Ti_3_AlC_2_ converted to Ti_3_C_2_T_x_ at room temperature in about five hours and yielded large mono- and bi-layer flakes with F-free terminations. The NH_4_^+^/NH_3_·H_2_O pair opens the interlayer galleries so that etching can propagate beneath the surface [38].

Chen et al., 2022, extend the concept with a mixed LiOH/LiCl electrolyte operated at about 5.0–5.5 V, where chloride extracts aluminum as AlCl_3_, hydroxide writes –OH terminations, and lithium intercalation widens the galleries so multilayers delaminate by simple sonication; the reported etching efficiency is approximately 92 percent and the strategy generalizes to other MAX precursors such as V_2_AlC [39].

A unifying motif threads these variants: chloride-assisted de-aluminization followed by hydroxylation and gallery opening. A simplified core reaction is as follows:

M_n+1_AX_n_ + 3e^−^ + 3Cl^−^ → M_n+1_X_n_ + AlCl_3_.

In lithium batteries and polymer composites, fluoride-free -O, -OH, and -Cl terminations improve wettability, adhesion, and SEI/CEI stability, while large few-layer flakes with widened galleries promote ion transport and fast charging [40,41]. These routes are compatible with aqueous, single-bath processing; for example, LiOH/LiCl etching followed by sonication aligns well with polymer-friendly manufacturing flows [41].

Set against these strengths, the process window is narrow. Tight control of time, voltage, and acidity is required to avoid over-etching, which converts the outer MXene into carbide-derived carbon and degrades conductivity and rate. Residual chloride in mixed terminations may need brief post-treatments, and chloride or alkaline media demand corrosion-resistant hardware together with careful hydrogen management. Operation at elevated cell voltages concentrates power and heat, so robust thermal and mass-transport design is essential. Scale-up is also non-trivial. Larger electrodes intensify field non-uniformity, mass-transport limits for Cl^−^/OH^−^ and for removal of AlCl_3_/AlOOH, and the challenges of heat and gas handling and fixture corrosion. These issues motivate engineered flow-cell or stacked-electrode architectures with pulsed or stepped potentials, active mixing and filtration, and in-line quality control [41,42].

#### 2.2.3. Molten-Salt Etching and Derivatives

##### Lewis-Acid Molten-Salt Etching Method

Li et al. (2019) first disclosed the Lewis-acid molten-salt approach in molten ZnCl_2_ as an element-replacement route. They showed that Al can be extracted from Ti_3_AlC_2_ through a transient A-site exchange to Ti_3_ZnC_2_ and that continued reaction in the melt yields Cl-terminated MXenes such as Ti_3_C_2_Cl_2_ and Ti_2_CCl_2_. This original study established an HF-free pathway to halogen-terminated MXenes and motivated mechanism-centric work on termination control and intermediate phases [43]. Li et al. (2020) then reported a broader Lewis-acid etching strategy that delivered high-rate, pseudocapacitive Li^+^ storage near 205 mAh g^−1^ and expanded applicability across MAX chemistries; together, these papers explain why molten-salt etching rapidly became a core synthesis alongside aqueous routes [26]. Kamysbayev et al. (2020) complemented this by showing that molten salts can program surface terminations, giving access to -Cl, -Br, and -I, which in turn tunes work function, interlayer spacing, and interfacial reactivity beyond the mixed -O and -OH and -F surfaces common to HF processes [44]. For downstream processing, Zhang et al. (2024) introduced an n-BuLi-free delamination based on LiCl in anhydrous solvents that preserves the designed -Cl termination, which is advantageous when casting films or performing in situ polymerization of composites [45]. On the application side, Shen et al. (2024) used Ti_3_C_2_Cl_2_ as a zincophilic, corrosion-moderating additive and host to homogenize Zn deposition in systems that pair naturally with gel polymer electrolytes, while MXene–fluoropolymer studies, such as Shepelin et al. (2021), support the compatibility of halogen-defined, fluorine-free MXenes with PVDF–HFP and PVDF–TrFE, including improved dispersion, β-phase promotion, and robust dielectric or percolation pathways that help cleaner SEI in gel and quasi-solid cells [46,47]. Mechanistically, the ZnCl_2_ pathway proceeds in two steps, with A-site exchange preceding halogenation:

(1) Ti_3_AlC_2_ + 1.5ZnCl_2_ → Ti_3_ZnC_2_ + 0.5Zn + AlCl_3_

(2) Ti_3_AlC_2_ + 1.5ZnCl_2_ → Ti_3_C_2_ + 1.5Zn + AlCl_3_

(3) Ti_3_C_2_ + Zn → Ti_3_ZnC_2_

A parallel CuCl_2_ system shows a Ti_3_CuC_2_ waypoint before conversion to Ti_3_C_2_Cl_2_, reinforcing the general picture of A-exchange followed by halogenation and helping define processing windows for different salts (Yan and Zhu et al., 2024) [48].

For device fabrication, LAMS offers HF-free chemistry that eliminates HF/LiF carryover and simplifies waste handling. Halogen terminations (-Cl, -Br, -I), broadened MAX compatibility, and delamination that preserves Cl together allow surface energy and electronic structure to be tuned to specific binders and electrolytes during scalable processing. These attributes fit well with fluorinated matrices such as PVDF–HFP and PVDF–TrFE. In such hosts, LAMS-derived MXenes disperse uniformly at low loading, reinforce the dielectric network, and support stable ion transport with cleaner SEI and CEI. The same halogen-defined surfaces promote fast pseudocapacitive storage in lithium-ion electrodes, and Ti_3_C_2_Cl_2_ in aqueous zinc cells serves as a zincophilic, corrosion-moderating scaffold that integrates effectively with gel polymer architectures. The trade-offs are largely engineering, since operation around 500–800 °C requires tight control of melt corrosion, transient M-MAX or metallic residues must be removed to prevent parasitic currents, and the lower hydrophilicity of halogen-terminated surfaces compared with O or OH often calls for solvent tailoring or a mild HF-free post-exchange to raise polarity without losing the advantages of LAMS.

##### Low-Temperature Hydrated Molten-Salt Etching

The earlier Lewis-acid molten-salt route removes the A-site in molten ZnCl_2_ at about 550 °C by oxidizing Al to Al^3+^, so that volatile AlCl_3_ drives out-diffusion and a Zn–MAX intermediate such as Ti_3_ZnC_2_ forms before conversion to the Cl-terminated MXene Ti_3_C_2_Cl_2_. In comparison, the low-temperature hydrated molten-salt route was developed to keep the synthesis HF-free, avoid high temperatures and corrosive chloride melts, and allow operation in air with milder work-ups and polymer-friendly surfaces [26]. In the hydrated route described by Pang et al., a dry blend of MAX (for example, Ti_3_AlC_2_) with LiCl and MgCl_2_·6H2O is heated in air at 150 °C for about 12 h so that the A-site is removed in a redox-controlled way while the MX scaffold remains intact [49]. As the temperature crosses about 117–118 °C, MgCl_2_·6H_2_O enters a dehydration and phase-change window that creates a semi-molten shielding layer, and Li+ increases ion mobility and intercalation, so etching proceeds at low temperature without harsh intercalators [49,50]. After cooling, warm water washing near 70 °C dissolves the salts and a short rinse with 2 M HCl in ethanol removes transient Mg and Al nanoparticles. Simple settle-and-redisperse cycles then give spontaneous delamination to single- or few-layer Ti_3_C_2_T_x_ with Cl/O/OH terminations; the product typically shows a downshift of the XRD (002) reflection and Cl 2p features in XPS, and the baseline yield is about 42% under these conditions [49]. The same principle extends to hard-to-etch MAX such as Cr_2_CT_x_, Nb_2_CT_x_, and Ti_2_NT_x_ while remaining fluoride-free and air-operable, so the main strengths are the mild temperature, the safer chemistry, the polymer-friendly O/OH/Cl surfaces, and the avoidance of aggressive intercalants. The corresponding limitations are a narrow thermal window around the hydrate transition, a critical dependence on LiCl whose absence causes surface-limited attack, a required acid cleanup step, and yields that cluster near 42% and are sensitive to handling, so tight control of salt composition and dwell time is preferred.

#### 2.2.4. Iodine-Assisted, Non-Aqueous Etching

Shi et al. reported the iodine-assisted, non-aqueous etching of Ti_3_AlC_2_ in anhydrous acetonitrile, producing ambient stable Ti_3_C_2_T_x_ with oxygen-rich terminations, thin-film conductivity near 1250 S cm^−1^, and supercapacitor performance around 293 F g^−1^ at 1 mV s^−1^. They also observed that an iodine-bearing intermediate forms first and then converts during post-treatment, which sets the foundation for this HF-free route [51]. Subsequent analyses summarized the same chemistry as a clean, fluoride-free pathway that begins by generating Ti_3_C_2_I_x_ and ends with O/OH-terminated MXene after work-up in acid or water, clarifying why this method improves ambient stability and avoids fluorine residues [46,52].

To center the mechanism, the non-aqueous iodine route proceeds through the formation of an iodide-terminated intermediate, followed by spontaneous or post-treatment exchange to oxygenated surfaces; a concise scheme drawn from the literature is

(1) Ti_3_AlC_2_ + (x+3)/2 I_2_ → Ti_3_C_2_I_x_ + AlI_3_

(2) Ti_3_C_2_I_x_ + x/2 O_2_ → Ti_3_C_2_O_x_ + x/2 I_2_

(3) Ti_3_C_2_I_x_ + x H_2_O → Ti_3_C_2_(OH)_x_ + x HI

Because iodine etching yields oxygen-rich, fluorine-free surfaces, Ti_3_C_2_T_x_ integrates naturally with polar matrices and polymer electrolytes. In PVDF-based dielectrics and piezoelectrics, MXene fillers can increase β-phase content, strengthen interfacial polarization, and improve dispersion, outcomes that support higher permittivity and self-poling behavior in PVDF–HFP and PVDF–TrFE films [53,54]. In gel or quasi-solid electrolytes and in PEO-type systems, the more hydrophilic O/OH terminations favor wetting, Li salt coordination, and homogeneous ion pathways. In electrochemical devices, these fluoride-free, oxygenated surfaces also mitigate HF/LiF artifacts and maintain high conductivity, which underpins the strong supercapacitor metrics reported for iodine-etched Ti_3_C_2_T_x_. The advantages are the HF-free workflow that improves safety and waste handling, the generation of oxygen-rich terminations that enhance hydrophilicity and polymer compatibility, and the good ambient stability and conductivity of the resulting flakes, which translate into robust films and electrodes. The limitations are mainly practical. Operators must handle iodine and AlI_3_ under dry conditions and perform a subsequent acid or aqueous step to delaminate and convert I terminations. Residual iodide can persist and therefore requires thorough washing. Etching can be diffusion-limited for thick particles, and the generality across all MAX chemistries is still uncertain.

#### 2.2.5. Photo-Fenton Soft-Chemistry Etching

A green, HF-free path to MXene uses light to drive a photo-Fenton cycle in Fe/H_2_O_2_ under mildly acidic conditions. Reactive oxygen species weaken Ti–Al bonds in Ti_3_AlC_2_, generate local OH^−^, dissolve Al as a soluble species, and leave O/OH-rich Ti_3_C_2_T_x_ that can be used directly as an electrochemically active host, as demonstrated for Li–S batteries [55]. Recent overviews of sustainable MXene synthesis place this soft-chemistry route alongside other HF-free methods and emphasize benign waste handling and compatibility with flexible devices [56].

The HF-free, light-driven photo-Fenton route generates O/OH-terminated Ti_3_C_2_T_x_ that has already functioned as a sulfur host in Li–S cells, so it connects naturally to practical battery use [55]. In polymer energy storage, this hydrophilic surface chemistry promotes good dispersion and strong interfacial coupling with polar or fluoropolymer matrices such as PVDF–TrFE, which supports water-based casting and printable MXene–polymer composites suited to flexible, solution-processed devices [46]. The main advantages for polymer systems are greener chemistry with simpler waste handling and, at the materials level, improved dispersion and adhesion that can translate into better Li^+^ transport in solid or gel polymer electrolytes. The main limitations are technical but manageable: residual iron or oxidants from the photo-Fenton cycle must be removed to avoid radical-driven side reactions during polymer processing, and oxidation of Ti_3_C_2_T_x_ toward TiO_2_ must be controlled to preserve conductivity; both points call for careful purification, storage, and processing protocols.

#### 2.2.6. Chemical Vapor Deposition–Bottom-Up MXene Growth

Bottom-up MXene growth by chemical vapor deposition was first demonstrated by Wang et al., 2023, who built Ti_2_CCl_2_ and Ti_2_NCl_2_ directly from vapors into vertically aligned carpets that expose dense edge planes and deliver excellent lithium intercalation, with wafer-scale control of thickness and orientation [57]. Gas-phase refinements followed: Xiang et al., 2024, clarified how to tune the chemistry for performance, and Yue et al., 2024, achieved one-step, few-layer, single-phase Ti_2_NCl_2_ and Ti_2_CCl_2_ while mapping activation and degradation pathways during growth [58,59]. At the mechanistic core, halide-assisted reactions nucleate carbide or nitride layers on metal surfaces and repeatedly refresh the reaction front, so sheets buckle upward and self-assemble into vertical carpets. A simple stoichiometry captures the idea: Ti foil plus TiCl_4_ plus CH_4_ yields Ti_2_CCl_2_ and HCl, while Ti foil plus TiCl_4_ plus N_2_ yields Ti_2_NCl_2_.

Chemical vapor deposition directly produces continuous, atomically thin transition-metal carbides and nitrides with precise control of thickness and phase and with very low sheet resistance, which enables wafer-scale patterned conductors and catalytic interlayers without the use of HF [57,60]. These strengths come with practical costs since growth demands a high thermal budget on metal-foil catalysts, film transfer with PMMA can leave cracks or polymer residues, and the as-grown surfaces are comparatively unfunctionalized and therefore wet polymers poorly until a brief post-activation or grafting step is applied [60,61]. Taken together, the most logical use in polymer energy storage is to deploy CVD-grown MXenes as laminated current collectors or engineered interlayers on PVDF- or PEO-based membranes and on gel or solid polymer electrolytes once the surface has been oxygenated or functionalized, which improves adhesion, strengthens interfacial polarization, and preserves continuous ion pathways at the contact. By contrast, when the goal is slurry-mixed polymer composites that depend on colloidal dispersion and anion anchoring throughout the bulk, fluoride-free etched O/OH-rich flakes usually provide a simpler and lower-cost route.

#### 2.2.7. Mechanochemical Fluoride-Free Synthesis

A fluoride-free, solvent-lean mechanochemical route provides a practical counterpart to HF methods by coupling chemistry and mechanics in a near-dry ball mill. Xue et al. (2020) showed that co-milling Ti_3_AlC_2_ with tetramethylammonium hydroxide and LiCl enables in situ hydroxide etching alongside defect-assisted delamination, yielding porous, oxygen-terminated Ti_3_C_2_T_x_ with strong lithium-ion performance [62]. Under high-energy milling, shear and impact open fresh fracture planes, hydroxide converts Al to soluble aluminate with hydrogen evolution, minimal water sustains the reaction, and Li^+^ promotes interlayer sliding and charge redistribution, so MAX transforms to MXene without fluoride. The same defects that speed etching also ease delamination, producing high-surface-area Ti_3_C_2_T_x_ rich in O/OH terminations that improve electrolyte wetting and ion transport. As Iravani et al. (2024) and Ljubek et al. (2023) note, outcomes depend on milling energy and time, ball-to-powder ratio, the identity and loading of the base and salt, controlled water content, jar and ball materials, and atmosphere; together, these tune flake size, defect density, and termination balance while keeping the process fluoride-free [63,64]. Advantages include elimination of HF and bifluorides, low liquid use with simpler wastes, compatibility with scalable mills, creation of high-area architectures, and hydrophilic surfaces that integrate well with gel or solid polymer electrolytes and composite electrodes. Practical limitations include possible contamination from jars or balls and less precise termination control than halogen-programmed routes. There is also an oxidation risk during work-up, along with the need for thorough washing and the potential for variability in yield or phase purity.

**Figure 1:**
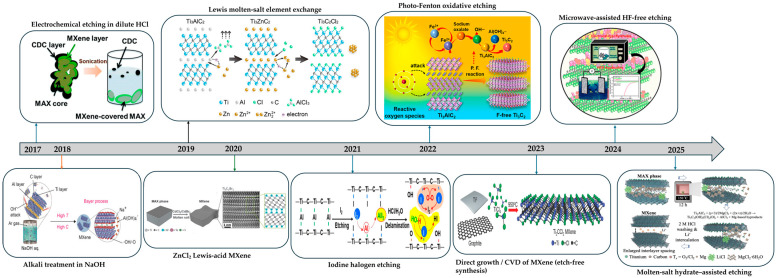
HF-free MXene synthesis milestones. Reproduced with permission from Ref. [37] (Royal Society of Chemistry, 2017); Ref. [25] (Wiley-VCH, 2018); Ref. [43] (American Chemical Society, 2019); Ref. [44] (AAAS, 2020); Ref. [51] (Wiley-VCH, 2021); Ref. [55] (American Chemical Society, 2022); Ref. [57] (AAAS, 2023); Ref. [35] (Royal Society of Chemistry, 2024); Ref. [49] (Wiley-VCH, 2025).

In summary, HF-based routes such as direct HF, in situ HF from LiF with HCl, and bifluorides are still the fastest and most established ways to remove the A layer from MAX phases. They are simple and scale well but demand strict safety controls, create fluoride-bearing waste, and often leave F-rich surfaces that reduce hydrophilicity and complicate interphases. Green routes such as alkali or acid hydrothermal (including salt-assisted or microwave variants), electrochemical etching, Lewis-acid molten salts, iodine-assisted non-aqueous etching, photo-Fenton chemistry, low-temperature hydrated salts, and mechanochemical milling instead aim for fluoride-free handling and steer terminations toward O or OH, which improves wettability, polymer adhesion, and SEI stability but requires tighter process control and careful residue removal. In polymer processing, these chemistries enable water-based inks, uniform casting with PVDF, PAN, PEO, and hydrogels, and easier gallery infiltration for continuous ion pathways. In energy storage, they support cleaner interfaces, lower interfacial resistance, higher rate capability, and longer cycle life, while halogen-programmed products align with fluorinated binders and can be post-exchanged to O or OH for hydrophilic systems. Table 1 summarizes synthesis routes, key features with concise pros and cons, and typical surface terminations, and Figure 1 charts the milestone progression across HF-free MXene syntheses.

## 3. MXene Property Pillars for Polymer LMB and Li–S Systems

MXenes pair metallic or semi-metallic transport with termination-rich layered surfaces, which in polymer electrolytes, interlayers, binders, and hosts govern interfacial continuity, coupled ion–electron pathways, and thermo-mechanical stability under realistic LMB and Li–S conditions. Because synthesis presets the termination ensemble, green routes become part of the property map as well as the safety case. Electrochemical and hydrothermal methods enrich -O and -OH and open water or alcohol processing windows, while Lewis-acid molten-salt routes yield Cl- and O-dominated surfaces that wet fluoropolymers and ionogels predictably in low-polarity media. HF etching typically leaves F together with -O and -OH and can be leveraged to seed LiF but carries handling hazards and narrows processing choices. This route-to-termination-to-processing link recurs across these four levers and ties polymer selection to device format.

### 3.1. Terminations and Wetting

Ti_3_C_2_T_x_ MXenes bear abundant polar terminations (-O, -OH, -F, etc.) that make their surfaces intrinsically hydrophilic and lithophilic [68,69]. In lithium-facing skins and polymer electrolytes, O- and OH-rich sheets from electrochemical or hydrothermal synthesis couple naturally to ether- or nitrile-based matrices and to hydrogels, lowering interfacial impedance and promoting uniform electrolyte uptake [29,30,70,71]. In sulfur electrodes and separator-like coatings, the same polar sites chemisorb lithium polysulfides, so shuttle is curtailed while wetting remains robust [72,73,74]. Molten-salt Ti_3_C_2_T_x_, typically Cl- and O-dominated, wets fluoropolymers and ionogels more reliably, which is valuable around nonpolar binders or at higher-voltage windows near the sulfur side. HF-derived F-rich surfaces can assist LiF formation at the anode, yet fluoride-free terminations reduce corrosive residues and better support water or alcohol dispersion without sacrificing film uniformity. For reproducibility, termination populations by XPS should be paired with contact-angle measurements under controlled humidity so that programmed polarity can be linked to impedance in LMB and ESR in Li–S [75].

### 3.2. Two-Dimensional Ion Pathways and Spacing

The layered architecture creates quasi-two-dimensional galleries that act as ion corridors. When interlayer spacing d(002) is stabilized or widened with benign intercalants or polymeric pillars, through-thickness tortuosity falls, room-temperature ionic conductivity at fixed thickness rises, and Li^+^ flux becomes more uniform in LMB membranes and interlayers [76,77,78,79]. In Li–S interlayers and MXene-rich hosts, stabilized galleries increase electrolyte access to catalytic sites and shorten diffusion paths for polysulfide conversion, which improves areal utilization and rate retention at practical loadings. Green synthesis supports this lever by limiting corrosive residues and surface damage that would otherwise collapse galleries during drying, making spacing control more repeatable. Electrochemical or hydrothermal Ti_3_C_2_T_x_ disperses in water or alcohol with short dwell times and compatible antioxidants, which slows oxidation and keeps d(002) close to the designed value until curing. Molten-salt Ti_3_C_2_T_x_ pairs naturally with low-polarity binders and ionogels that help maintain gallery order [29,30,45,71,80]. Structure–transport mapping should combine humidity-controlled XRD or SAXS for spacing with temperature-dependent impedance so gains in conductivity can be traced to channel geometry rather than to uncontrolled interfacial reactions.

### 3.3. Interfacial Reactivity and Interphase Control

At reactive contacts, terminations modulate nucleation barriers, local fields, and near-surface solvation, so interphases form differently depending on the route. On lithium, Ti_3_C_2_T_x_ interlayers and ultrathin skins distribute current and favor compact LiF-rich yet ion-permeable interphases that raise critical current density and stabilize lean electrolyte formats. In fluoride-free Ti_3_C_2_T_x_, LiF arises mainly from controlled salt decomposition rather than residual HF, a shift that often yields smoother morphology and lower charge-transfer resistance at comparable conditions [70,71,81,82]. O- and OH-rich surfaces further tune the local solvation environment and promote uniform Li^+^ desolvation, while Cl- and O-dominated surfaces from molten salt tend to remain stable in low-polarity ionogels and fluoropolymer binders. On the sulfur side, Ti_3_C_2_T_x_ anchors and catalyzes polysulfide intermediates so that redox conversion accelerates, and shuttle is suppressed, especially when the termination ensemble is matched to polar domains in the binder and to the chosen electrolyte. These interfacial gains should be documented with standard Coulombic efficiency protocols, impedance evolution, and post-cycling spectroscopy or microscopy to allow fair comparisons across chemistries and routes.

### 3.4. Mechanics and Heat Management

Plate-like MXenes reinforce polymers at modest loading while adding efficient in-plane heat-spreading pathways. In LMB polymer electrolytes and interlayers, the higher storage modulus resists filament penetration under realistic current density and stack pressure, and elevated thermal conductivity reduces hot spots during fast charge. In Li–S electrodes and interlayers, the same thermal pathways temper Joule heating at high rate and help preserve interfacial contact over long cycling. Green processing strengthens this lever by preserving flake aspect ratio and minimizing defect-induced embrittlement. Electrochemical and hydrothermal Ti_3_C_2_T_x_ enable aqueous or alcohol routes where brick-and-mortar reinforcement is achieved at lower filler fraction, while molten-salt Ti_3_C_2_T_x_ pairs with ionogels and ester media that sustain thermal stability at elevated power [29,30,45,71,80,83]. Mechanical and thermal metrics should be co-reported on the same specimens with matched thickness and humidity, so reinforcement and heat spreading are not confounded by unintended electronic percolation in electrolyte-rich regions.

MXene–polymer performance in lithium-metal and lithium–sulfur batteries is governed upstream by route-programmed surface terminations and the layered lattice. Matching the termination palette to the polymer environment secures continuous wetting and low interfacial impedance on lithium-facing layers while providing strong anchoring of polysulfides at the cathode. Stabilizing and widening interlayer spacing creates two-dimensional ion corridors that reduce tortuosity, raise ionic conductivity at fixed thickness, and equalize Li^+^ flux. Termination-guided interfacial reactivity promotes compact, ion-permeable interphases on lithium and catalyzes polysulfide conversion, which increases critical current density and rate retention. Plate-like flakes add modulus and in-plane heat spreading so thick films remain shape-stable and cooler under high power.

Two moderators cut across all pillars: electronic networks should remain below the percolation threshold in electrolyte-rich regions, and surface chemistry must be preserved by controlling oxidation and humidity from dispersion through curing. Table 2 consolidates these points into a checklist that links descriptors and methods to the device metrics to be reported and the practical controls to apply.

## 4. Synthetic Methods of MXene–Polymer Composites

MXene–polymer composites are prepared by a variety of techniques that dictate the filler–matrix interfaces and final morphology. Common strategies include simple solution mixing (with film casting), in situ polymerization, covalent grafting, layer-by-layer (LbL) stacking, and electrospinning. Each approach differs in how MXene flakes are integrated into the polymer, affecting dispersion, interfacial bonding, and mechanical properties. These composites have been explored as binders, separators, or electrolyte hosts in Li-metal and Li–S batteries. Below, we compare these methods in terms of processing, interface interaction, and structure control.

### 4.1. Solution Blending and Film Casting

Solution blending (often followed by casting or coating) is a straightforward physical-mixing route in which exfoliated MXene (for example Ti_3_C_2_T_x_) is first dispersed in a solvent, typically water, to leverage MXene hydrophilicity; then, a polymer solution is introduced and mixed by stirring or sonication to obtain a homogeneous dope that is dried to form a composite film or membrane [18,84,85]. Common polymer choices include water-soluble systems such as Polyvinyl alcohol (PVA), Polyethylene oxide (PEO), and PDDA; when the target matrix is a polar but water-insoluble polymer such as PVDF or PAN, researchers first transfer the dispersion by solvent exchange and then blend. A representative case is Ti_3_C_2_T_x_ added to PEO–LiTFSI via an aqueous route, which yields a uniform polymer electrolyte film with increased amorphicity [86]. Mechanistically, the process consists of dispersing MXene within the polymer solution, mixing thoroughly to limit restacking, and removing the solvent to consolidate a coherent film. Casting and coating are carried out by spray coating, spin or blade coating, drop casting, or vacuum filtration of the MXene–polymer solution, and parameters such as viscosity and shear govern thickness control and flake alignment. Its main limitations are that interfaces are predominantly noncovalent unless the polymer is functionalized, which can restrict adhesion; films can suffer from loss of flatness or toughness due to flake restacking and may become brittle at high filler loadings; and thickness control often requires post-processing such as calendaring to achieve the desired density [87,88].

### 4.2. In Situ Polymerization

In situ polymerization involves chemically polymerizing monomers in the presence of MXene flakes so that the polymer network forms around or on the filler; in practice, MXene is dispersed in a liquid monomer or prepolymer under mild conditions and polymerization is initiated thermally or by oxidants or redox agents to yield a composite in which chains grow near or from the MXene surface and enhance interfacial contact [89]. Typical implementations include oxidative polymerization of anilines or pyrroles to generate PANI or PPy coatings directly on MXene, and free-radical polymerization of acrylates to build composite electrolytes or conductive binders with well-distributed Ti_3_C_2_T_x_ flakes [90,91,92]. The mechanism is straightforward: MXene is mixed with monomers or prepolymers, initiation proceeds in situ, and dense chain formation at the MXene surface improves compatibility and dispersion as the matrix gels or solidifies [18,89]. The main challenges are the compatibility of reaction conditions with MXene stability, since harsh oxidants, extreme pH, or high temperature can degrade the flakes, the usable monomer set is narrower than in blending, process control is more complex, and the residual monomer or initiator must be removed carefully after gelation or curing [93,94].

In practice, two distinct in situ modes are relevant for MXene–polymer systems in batteries. In bulk curing schemes, the MXene-containing monomer or oligomer is polymerized in a free-standing film or porous scaffold before cell assembly. The resulting MXene–polymer network fills the electrolyte or separator volume and mainly governs ionic conductivity, mechanical strength, and electrode wetting [95,96]. By contrast, interfacial in situ polymerization is performed after a low viscosity MXene/monomer layer has been applied onto a current collector or Li metal. Polymerization is then confined to a thin interphase so that the MXene-reinforced polymer grows from the surface outward and becomes an integral part of the SEI or CEI. This distinction is particularly important in Li-metal cells, where only the interfacial route directly constructs an artificial MXene–polymer SEI on Li [97,98,99].

### 4.3. Surface Grafting (Grafting-to and Grafting-from)

Surface grafting uses covalent chemistry to anchor polymers onto MXene surfaces and create dense polymer “brushes” that tightly couple the filler and matrix; in a grafting-from route, the MXene is first endowed with initiator or reactive sites and monomers are polymerized directly from the surface, whereas in grafting-to, preformed polymer chains carrying functional end groups are chemically coupled to MXene terminations such as –OH or –NH_2_ to yield flakes uniformly coated with tethered chains [100,101]. Mechanistically, silane and diazonium couplings are well-established for Ti_3_C_2_T_x_ and enable the attachment of polymers such as poly(ethylene glycol) or polymethacrylates, while surface-initiated routes can grow acrylic chains directly from MXene and polydopamine coatings can form by dopamine self-polymerization on Ti_3_C_2_T_x_ under mild alkaline conditions [102,103,104,105,106]. The key advantage is the creation of covalent or strongly tethered interfaces that enhance compatibility, suppress interfacial slippage, and distribute stress, often delivering superior cohesion and fracture resistance compared with non-grafted blends. The main drawbacks are synthetic complexity and limited scalability, since multi-step functionalization and careful control of graft density are typically required, and the accessible chemistry window is constrained by MXene surface reactivity and polymer solubility, which can restrict monomer and solvent choices during processing.

### 4.4. Layer-by-Layer (LbL) Assembly

Layer-by-layer assembly builds MXene–polymer composites by alternating the deposition of thin layers of oppositely charged components so that a substrate is sequentially dipped or sprayed into a polycation solution and an anionic MXene dispersion with rinsing between steps, which yields a stratified multilayer film with angstrom-to-nanometer control over thickness and composition. The mechanism relies on the electrostatic attraction between negatively charged MXene sheets and a positively charged polymer, and cyclic adsorption followed by rinsing adds successive bilayers, while hydrogen bonding, π–π interactions, and van der Waals forces further stabilize the stack and reduce defect density [107,108]. The main advantages are atomic-scale architectural control, uniform MXene coverage that minimizes agglomeration, and tunable thickness, pore structure, and porosity by adjusting the number of bilayers, which can produce good mechanical integrity when charge complementarity is designed appropriately [107,109]. The primary drawbacks are low throughput because each layer requires an adsorption and rinse cycle, suitability mainly for thin films rather than bulk composites, a polymer palette that is biased toward polyelectrolytes such as PSS or chitosan unless MXene is chemically modified to adjust charge, and challenges in scaling beyond laboratory formats [108,110].

### 4.5. Electrospinning

Electrospinning integrates MXene into nanofibrous polymer mats by ejecting a polymer solution or melt containing dispersed MXene through a needle under a high-voltage electric field. The charged jet thins into nano- to microfibers and deposits as a nonwoven layer on a grounded collector, embedding two-dimensional flakes within or on the fibers [111,112]. Mechanistically, polymers such as PAN, PVDF, or PI are dissolved with MXene to obtain a viscous spinning dope. Under about 10–30 kV, the jet stretches, the solvent evaporates, and MXene becomes locked into the interconnected fiber network, while collector motion can align the fibers and bias flake orientation [113,114,115]. This approach yields a three-dimensional network with high porosity and continuous pathways for ion percolation. The fibrous mesh gives good mechanical flexibility and can promote favorable polymer crystalline phases or orient MXene flakes, as seen in PVDF fibers that favor the electroactive β phase. MXene addition also enhances fiber wettability and strength, consistent with reports on PI/MXene mats [116,117,118]. The main limitations are that electrospinning typically yields thin mats on the order of tens to a few hundred micrometers so thicker separators require stacking or densification, many formulations rely on volatile or toxic solvents, uniform MXene dispersion is critical to avoid phase separation and jet clogging, and additional binders or post-treatments may be needed for robust electrode architectures.

**Figure 2:**
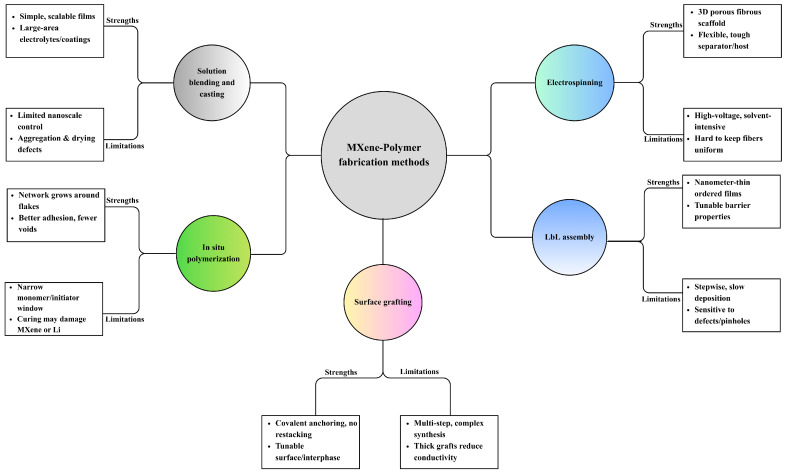
MXene–polymer fabrication methods and their main strengths and limitations.

In summary, MXene–polymer composites are commonly prepared by five complementary methods, as summarized in Figure 2. Solution blending with film casting gives simple and scalable membranes or coatings that are suitable for solid and gel polymer electrolytes or separator-like layers, where dispersion and drying govern ion transport uniformity, interfacial contact, and mechanical integrity. In situ polymerization lets the polymer network grow around the flakes and at buried interfaces, which improves adhesion, reduces voids, and stabilizes gel or solid electrolytes and tough binders, but the usable monomer and curing windows are more limited. Surface grafting, either grafting-to or grafting-from, creates covalent brushes that keep flakes separated, tune compatibility, and build durable interphases in high-loading electrodes, at the expense of additional synthetic steps. Layer-by-layer assembly stacks charged species in ordered nanometric films that act as ultrathin separators or interlayer barriers, offering precise control over thickness and tortuosity but relatively low throughput for large-area production. Electrospinning forms porous nanofiber mats with embedded or aligned flakes, providing wettable, breathable scaffolds for separators and hosts that can accommodate large volume changes, while fiber uniformity and solvent recovery need to be controlled. The following Table 3 compares these five methods in terms of process nature, dominant interface chemistry, morphology control, mechanical fit, scalability, and common pitfalls, and Figure 2 maps each route to its typical strengths and limitations.

## 5. From Terminations to Composites: Design Rules for MXene–Polymer Systems

The following sections translate route-controlled MXene terminations into practical design rules for MXene–polymer energy storage systems spanning batteries. Here, T_x_ denotes the surface termination ensemble on MXene flakes, and polymer matching means both interfacial complementarity with the backbone and compatibility with the processing medium that preserves chemistry and microstructure. Here, MXene-based polymer families are grouped into ether-rich hosts (PEO/PEG-based backbones), nitrile- and carbonyl-containing coordination-rich networks (PAN, polycarbonates, urethane/acrylate systems), fluoropolymers (PVDF and PVDF–HFP), hydroxyl-/carboxyl-/ester-bearing biopolymers (alginate, starch acetate, cellulose), and COF-type porous polymer frameworks. A light but explicit green thread is maintained: when performance is comparable, fluorine-free terminations and NMP-free processing are preferred. The discussion proceeds from a route-to-termination-to-polymer design map, through interfacial mechanisms, into processing and microstructure guidance, and concludes with compact rules and benchmarking to enable reliable, comparable, device-level metrics.

### 5.1. Purpose and a Route-to-Termination-to-Polymer Design Map

Designing MXene–polymer composites for Li-metal and Li–S batteries starts from the etching route, because it fixes the termination ensemble and thus which polymers and solvents are compatible. HF-free processes such as alkali, electrochemical, or Lewis-acid molten-salt etching usually give O/OH-rich surfaces [56]. These polar, hydrophilic MXenes bond strongly to ether- or nitrile-based polymers (e.g., PEO, PAN) and to waterborne systems, so they promote Li^+^ coordination and salt dissociation in polymer electrolytes [56,86]. By contrast, molten-salt and halogen-gas routes produce halide-rich terminations (-Cl, -Br, -I) that wet fluorinated polymers and ionogels (e.g., PVDF–HFP backbones) more predictably and support high-voltage stability. F-rich MXenes from HF routes can still be used but often require post-treatments or compatibilizers to avoid poor dispersion and disturbed interfacial chemistry in polar matrices [56].

A concise design map emerges as follows: choose an etchant class that yields terminations complementary to the target polymer and processing solvent. HF-free, “green” MXene routes (alkali, electrochemical, molten-salt, etc.) generate primarily O/OH/Cl terminations, which pair with aqueous or low-toxicity solvents and with polar matrices. For example, Pan et al. fabricated a PEO–LiTFSI polymer electrolyte with 2D Ti_3_C_2_T_x_ MXene by a green aqueous mixing method; the MXene’s hydrophilic surfaces suppressed polymer crystallinity and increased Li^+^ mobility [86]. In contrast, if using PVDF–HFP or PMMA binders (common in Li-metal battery electrolytes or Li–S cathodes), a Cl-rich MXene from molten-salt etching can improve wetting and form beneficial LiCl/LiF-rich solid–electrolyte interphases.

In summary: The synthetic route → T_x_ (surface chemistry) → polymer family mapping determines which polymer backbones and salts are most compatible (ether/nitrile with O/OH-rich MXenes; fluorinated/ionic liquid gels with halide-rich MXenes).

### 5.2. Evolution of Polymer Backbones in MXene-Based Battery Systems

Polymer backbones in MXene-based battery systems have evolved from simple linear hosts into architected networks that are deliberately co-designed with MXene to control ion transport, mechanics, and interfacial chemistry. Early studies mainly used linear PEO or PVDF/PVDF–HFP as solid or gel polymer electrolytes and binders, where the polymer served as a passive salt host and MXene acted as a high-surface-area filler. Incorporating small amounts of Ti_3_C_2_T_x_ into PEO suppressed crystallinity, increased segmental motion, and raised Li^+^ conductivity, while PVDF-type binders with MXene improved electronic wiring but still offered limited regulation of polysulfide shuttling or interphase formation [86]. These first-generation systems demonstrated that MXene could enhance transport and mechanical robustness, yet the backbone chemistry itself remained relatively inert.

As the field matured, the design shifted toward more polar- and coordination-rich backbones that interact directly with both Li^+^ and MXene surfaces. Nitrile-, carbonyl-, and urethane-bearing polymers such as PAN, polycarbonates, and urethane or acrylate networks provide strong dipole–ion and dipole–MXene interactions. In PEO–PAN composite electrolytes reinforced by LLZTO and Ti_3_C_2_T_x_, ether and nitrile groups coordinate Li^+^ while MXene terminations weaken the Li–polymer association and immobilize anions. This combination increases room-temperature ionic conductivity, improves modulus, and stabilizes Li‖Li cycling and Li-based full cells compared with simple PEO/MXene systems [119]. Similar polar backbones used as binders, separators, or interlayers help homogenize Li^+^ flux, buffer volume change, and moderate SEI or cathode–electrolyte interfaces in Li-metal and Li–S configurations [120]. More recently, crosslinked and quasi-solid networks have been introduced to decouple mechanical integrity from segmental mobility. PEGDA-based acrylate networks formed in situ inside PVDF–HFP, with MXene sheets embedded as conductive and ion-guiding planes, create three-dimensional polymer–MXene frameworks. Covalent junctions lock in dimensional stability, while plasticized segments and MXene terminations maintain fast ion transport. Representative quasi-solid electrolytes of this type reach conductivities in the 10^−3^–10^−2^ S·cm^−1^ range and sustain long-term symmetric metal cycling with suppressed dendrite growth and reduced overpotential [121]. This shows that tuning crosslink density and architecture is an effective way to balance toughness and transport in MXene-based electrolytes.

In parallel, biopolymer backbones have emerged as sustainable matrices that align with fluoride-free MXene synthesis and safety-oriented electrolyte design. Sodium alginate, starch acetate, and cellulose provide abundant carboxyl, hydroxyl, and ester groups that can coordinate Li^+^, hydrogen bond with MXene terminations, and immobilize soluble intermediates. A conductive crosslinked sodium alginate@MXene binder enables Li–S cathodes with very high volumetric capacity and high sulfur loading, by combining a mechanically robust hydrophilic network with a percolating MXene skeleton for electron and ion transport [122]. Starch acetate grafted to MXene forms an all-solid polymer electrolyte that matches or surpasses room-temperature liquid electrolytes in Li-ion cells, while cellulose-based solid electrolytes filled with 2D or inorganic fillers reach conductivities on the order of 10^−4^–10^−3^ S·cm^−1^ and support stable Li-metal cycling [123,124].

At the more ordered end of MXene-based polymer backbones, covalent organic frameworks (COFs) can be regarded as crystalline porous organic polymers with long-range order, permanent porosity, and designable functional sites [125,126]. When integrated with MXene into COF/MXene heterostructures, the COF lattice offers size-selective ion channels and polar binding sites, while Ti_3_C_2_T_x_ furnishes continuous electron pathways and mechanical support. In Li–S electrodes and interlayers, COF@MXene hosts can trap polysulfides, accelerate redox kinetics, and maintain high reversible capacity over extended cycling [127].

Taken together, these developments show a clear evolution from linear polyethers and fluoropolymers to polar copolymers, crosslinked networks, biopolymer matrices, and COF-type frameworks. Across electrolytes, binders, separators, and artificial interphases, polymer architecture, functionality, and MXene–polymer interfacial interactions have become key levers to tune ionic conductivity, electrochemical stability, and overall electrode performance in MXene-based battery systems.

### 5.3. Interfacial Chemistry and Polymer Matching

At the polymer–MXene interface, termination chemistry governs salt coordination, polymer structure, and polysulfide interactions. O/OH-terminated MXenes bond via H-bonds and acid–base contacts to ether/nitrile chains and hydrogels, effectively creating continuous polar domains. In a Li-metal battery polymer electrolyte, these interactions promote Li^+^-polymer solvation and disrupt local polymer crystallinity, increasing ionic conductivity. For example, adding a few weight percent of Ti_3_C_2_T_x_ to PEO-LiTFSI (via aqueous mixing) not only retarded PEO crystallization but also enabled a continuous Li^+^ conduction channel [86]. The functionalized MXene surface promotes salt dissociation, raising Li^+^ transport numbers. Similarly, in Li–S batteries, a composite gel or solid polymer electrolyte with polar segments will benefit from oxygenated MXene: the polar MXene surfaces adsorb and immobilize migrating Li polysulfides, mitigating the shuttle. Liu et al. showed that a PVDF–HFP/Ti_3_C_2_T_x_/LiTFSI gel electrolyte suppressed polysulfide migration and dendrite growth, enhancing anode-free Li–S cycle life [128].

By contrast, Cl- or O-terminated MXenes (from Lewis-acid routes) excel with fluoropolymers and ionogels. These hydrophobic matrices often operate at higher voltages, where a stable nonpolar interface is critical. Cl-terminated MXene surfaces introduce Li–Cl and Li–F domains in the SEI that are ionically conductive yet electronically insulating. For example, coating a PP separator with Ti_3_CNCl_2_ MXene (Cl-rich) dramatically improved electrolyte wettability and induced a LiF/LiCl-rich SEI on Li metal, yielding uniform Li deposition. Such MXene/polymer composites help sustain high-voltage operation by minimizing solvent decomposition and preventing dendritic hot spots. Moreover, MXenes with Lewis-acid surface dopants (e.g., Zn-MXene or Co/Ni doped MXene) can act as interfacial catalysts. In Li–S cathodes, single-atom Zn-doped Ti_3_C_2_ enhanced LiPS conversion kinetics: the Zn sites on green MXene lowered the activation barrier for Li_2_S↔Li_2_S_2_ and created strong LiPS binding, stabilizing sulfur redox. Likewise, fluorine-free (F-free) Ti_3_C_2_ (rich in O/OH) prepared by photo-Fenton etching stored extra electrons on its surface, boosting sulfur lithiation rates and polysulfide anchoring [56].

Three practical guidelines emerge:

(1) Ionic affinity alignment: Match polymer–salt solvation to MXene termination. Ether segments (–O–) in PEO coordinate Li^+^ and pair best with –O/OH-MXenes; fluoropolymer/ionic liquid systems pair better with halide-MXenes.

(2) Processing solvent compatibility: Polar MXenes (O/OH-rich) disperse well in water/alcohols, enabling aqueous or benign solvent processing. Nonpolar polymers (PVDF, silicones) require organic or ionic liquid media, consistent with MXene wetting from Cl terminations.

(3) Filler architecture control: Use low-to-moderate MXene loadings with good exfoliation. Overloading can create electronic percolation or brittleness. Often just a few wt% MXene yields large gains. For example, 1.5–3.6 wt% Ti_3_C_2_ in PEO raised ionic conductivity substantially. When through-thickness ion transport is rate-limiting, align flakes (shear-oriented films or porous scaffolds) to shorten ion paths. Vertically aligned MXENE channels have been shown to cut ion tortuosity in polymer films by 2× under similar conditions.

### 5.4. Processing and Microstructure That Preserve Chemistry

Maintaining the intended MXene termination ensemble through processing is critical. First, solvent and safety: Prefer benign media. Pan et al. demonstrated that water enables the homogeneous mixing of Ti_3_C_2_T_x_ in PEO/LiTFSI, obviating toxic NMP and preserving –O/OH surfaces. Fluoropolymer binders (PVDF–HFP) can be processed from PVDF latex or coated from mild organic solvents if MXene is suitably terminated. In all cases, minimizing oxygen and moisture exposure during processing limits unwanted oxidation: freshly made MXene dispersions should be used quickly or stabilized (e.g., with sodium ascorbate), and films should be dried under inert or controlled humidity [71,129,130]. Notably, Cl-rich MXenes withstand humid environments better than F-rich ones, an advantage for green routes. In practice, casting the composite in air should be swift and films annealed only briefly if necessary.

Second, oxidation control: Ti_3_C_2_T_x_ can gradually oxidize in water/air, degrading conductivity and interfacial function. Shorten aqueous processing times and use antioxidants in inks. For example, adding trace reducing agents or Li salts can suppress oxide formation. After casting, a mild vacuum or mild heat step can remove residual interlayer water without triggering oxidation. These precautions retain surface chemistry and conductivity, which is vital for the Li-metal interface.

Finally, microstructure engineering: Ensure MXenes are delaminated and well-dispersed. Use shear mixing, ultrasonication, or benign intercalants (e.g., LiCl) to exfoliate flakes. A homogeneous 2D dispersion yields uniform ion pathways. Fabrication methods should match device geometry: solution casting or doctor-blading is versatile for flat solid membranes (PEO or PVDF composites), while electrospinning or 3D printing can embed aligned channels or porous scaffolds. For Li-S, layered sulfur/MXene composites or coaxial fibers can both trap LiPS and facilitate ion transport. Wherever possible, introduce ordering: even modest flake alignment in-plane (via shear) or out-of-plane (via magnetic field or directional freeze-casting) reduces tortuosity [131,132,133]. For example, the sequential “layer-by-layer” assembly of MXene/PVA hydrogels produced dense, reinforced electrolytes with high ionic conductivity and mechanical strength. Ultimately, specify processing details in reports: solvent system, inert atmosphere or not, film thickness, and humidity.

### 5.5. Compact Rules, Benchmarking, and a Failure-Mode Playbook

A concise set of rules bridges MXene chemistry and device metrics in Li-metal and Li–S cells.

Rule 1: Polar conduction networks. Assemble the composite so that polar pathways span the film thickness. In Li-metal solid or gel electrolytes (PEO, PMMA, ionogel), a few wt% MXene with -O, -OH terminations can interconnect ether segments into continuous channels. In Li–S cathodes or electrolytes, MXene surfaces should adsorb Li^+^ and LiPS, effectively integrating sulfur conversion domains. The result should be room-temperature conductivities > 10^−5^–10^−4^ S/cm with MXene assistance, without compromising film integrity.

Rule 2: Mechanics vs. softening. Suppress polymer crystallinity only as much as is needed to boost conductivity, but not so far that the electrolyte or binder loses rigidity. For Li-metal electrolytes, the storage modulus should remain above the threshold to block dendrite penetration (typically >MPa range). Composite theory and experiment both suggest co-reporting conductivity and modulus, e.g., a PEO composite whose crystallinity is partly disrupted by MXene can double ionic transport while still being mechanically stable. In Li–S cathodes, the binder modulus must support cathode volume changes; moderate MXene can toughen the network (via interfacial H-bonds) while providing extra ion paths.

Rule 3: Controlled percolation. Leverage MXene’s conductivity only when needed. Unintentionally forming an electronic percolation network (especially in a polymer electrolyte) risks internal short circuits or electrolyte leakage current. Keep MXene below the percolation threshold for electrons. If electronic conduction is beneficial (e.g., a thin cathode or current collector), then use aligned or graded architectures: for instance, embed MXene-rich layers near current collectors but not spanning the entire separator. In Li–S, multi-functional separators can be graded: one side rich in MXene for LiPS adsorption, the other lean to limit shorting. Empirically, vertically aligned MXene channels dramatically reduced tortuosity and allowed high conductivity at low loadings.

Rule 4: Measurement transparency. Always report ionic conductivity normalized by thickness (S/cm) at given T and RH, and specify the cell geometry and transference measurement method. For Li-metal cells, report the Coulombic efficiency or critical current density at a fixed area, noting stack pressure. For Li–S cells, report areal sulfur loading and capacity at fixed C-rate, ESR, and cycling stability. Transference numbers (t^+^) should use a well-defined protocol; the Bruce–Evans method is common but note if any corrections for polarization were applied.

Rule 5: Chemistry matching for stability. Choose MXene terminations consistent with the salt anion and voltage window. Cl/OH-rich surfaces pair well with PF_6_^−^ or TFSI^−^ salts in ether matrices (low-voltage Li-metal). For Li–S, a polymer electrolyte with LiTFSI or LiFSI will benefit from O/OH MXene that attracts Li^+^ while trapping LiPS. If fluorinated surfaces are unavoidable (HF-etch routes), mild post-functionalization (e.g., replacing –F with –OH by heating) or the use of compatibilizers (ionic liquids) can restore compatibility with polar polymers.

Benchmarking checklist: For each composite report the following: MXene source and etchant class; XPS-derived T_x_ (with fitting details); XRD d(002) spacing; flake size; solvent system (note “NMP-free” if applicable); processing atmosphere; membrane/electrode thickness; testing humidity; ionic conductivity (25 °C, 40 °C, activation energy); tLi^+^ measurement method; mechanical modulus; and, for Li-metal anodes, critical current density under specified pressure; for Li–S, areal capacity and capacity retention over >100 cycles at specified C-rate. When multiple routes perform similarly, prefer HF-free etching and NMP-free processing to minimize hazards.

Failure modes: If resistance rises or color darkens, suspect MXene oxidation—counter with inert handling, shorter aqueous steps, or antioxidants. If Li plating becomes non-uniform or dendritic, ensure MXene alignment/coating is uniform, or add a protective MXene–polymer SEI (e.g., MXene@PMMA) to homogenize ion flux. Weak polysulfide confinement (in Li–S) appears as rapid capacity fade; mitigate by enhancing MXene dispersion or using dopants (Zn, Co) to increase LiPS binding. If the film softens too much (modulus drops with conductivity), reintroduce crosslinking or a stiffer co-polymer to balance rigidity. In all cases, including basic processing metadata (solvent, atmosphere, drying, flake size, thickness) help diagnose issues and replicate results.

## 6. Application of MXene–Polymer Composites in Lithium Batteries: HF-Based MXene Focus

This section focuses on MXene–polymer composites in which the MXene is produced by fluoride-containing etching routes, yielding a characteristic -F, -O, -OH termination profile that governs wetting, ion selectivity, and interfacial chemistry, often favoring LiF formation under reducing conditions [17,134]. Within this HF-based scope, MXene–polymer hybrids provide a versatile two-dimensional framework that simultaneously addresses transport limitations, interfacial losses, and mechanical–thermal fragility. When polar, wettable, HF-etched MXene surfaces are coupled with ionically conductive yet electronically non-percolating polymer matrices, room-temperature ionic conductivity and effective Li^+^ transference increase, solid–solid contact becomes more intimate and durable with lower interfacial impedance and a LiF-inclined interphase, and toughness is enhanced through in-plane heat spreading that resists local hot spots and morphological instability [17,18]. Performance follows from co-design rather than any single ingredient, which means polymer and salt selection, the HF-derived termination profile and MXene identity, mesoscale architecture such as dispersed flakes, aligned lamellae, or ultrathin coatings, MXene loading kept below electronic percolation, and processing routes that lock in dispersion and adhesion must be chosen coherently [18]. With this perspective, the next subsections apply the same HF-based material logic to two battery systems, namely lithium-metal batteries and lithium–sulfur batteries, balancing transport, interfacial, and stability trade-offs while highlighting practical design levers specific to HF-etched MXene surfaces.

### 6.1. Lithium-Metal Batteries (LMBs)

In lithium-metal batteries, MXene–polymer hybrids are leveraged to boost room-temperature Li^+^ transport, lower interfacial resistance, and maintain electrode integrity under high C-rates. The key is pairing polar, wettable MXene surfaces with ion-conductive yet electronically non-percolating polymer matrices so solid–solid contacts remain intimate and durable over cycling. In practice, the same material logic is deployed in three places: within the electrolyte to encourage continuous Li^+^ pathways, as a thin interlayer or separator coating to ease contact and reduce resistance, and inside the electrode binder to keep the particle network coherent at high rates.

#### 6.1.1. MXene–Polymer Electrolytes in Lithium-Metal Batteries

**Figure 3:**
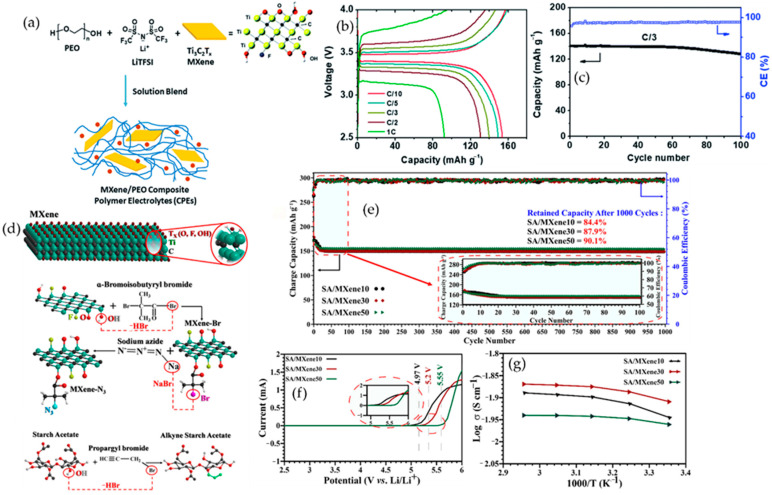
(**a**) Schematic of PEO/LiTFSI with few-layer Ti_3_C_2_T_x_ showing dispersion, reduced crystallinity, and Li^+^ pathway formation; (**b**) Rate performance of LFP∥PEO/LiTFSI/MXene∥Li across increasing C-rates; (**c**) Cycling stability of LFP∥PEO/LiTFSI/MXene∥Li at a fixed rate; (**d**) Click-grafting scheme and microstructure of starch-acetate-grafted MXene quantum dots; (**e**) Long-term full-cell cycling of SA/MXene solid polymer electrolytes; (**f**) Electrochemical stability window and ionic conductivity characteristics of SA/MXene electrolyte; (**g**) Temperature-dependent ionic conductivity of the prepared SPEs. Panels (**a**–**c**) reproduced with permission from Ref. [86] (Royal Society of Chemistry, 2019); panels (**d**–**g**) from Ref. [123] (Wiley-VCH, 2025).

MXene–polymer electrolytes have evolved from simple Ti_3_C_2_T_x_ dispersions toward surface- and network-guided designs that link fast ion transport to stable lithium interfaces. In the dispersion baseline from Pan et al., a small MXene fraction in PEO/LiTFSI reduces crystallinity enough to open Li^+^ pathways; the blend concept and expected microstructure are shown in Figure 3a and set the processing reference for later designs. Rate performance follows this mechanism: Figure 3b records C/10 to 1C profiles with capacities around 150–140 mAh g^−1^ at C/10–C/3 and about 90–95 mAh g^−1^ at 1C, while Figure 3c shows C/3 cycling for 100 cycles with Coulombic efficiency near 99–100 percent, confirming practical stability at low MXene loading [86]. Building on this baseline, Xu et al. convert MXene from discrete flakes into a continuous PAN/Ti_3_C_2_T_x_ scaffold that is back-filled with PEO–LLZTO; this lifts conductivity into the 10^−4^ S cm^−1^ class and sustains symmetric Li∥Li operation even near −2 °C, which addresses the transport and mechanical limits of dispersion-only films without sacrificing interface quality [119]. Shifting from architecture to chemistry, Qian et al. graft an ionic liquid onto MXene to prevent restacking, promote salt dissociation, and anchor TFSI^−^ so conductivity and the lithium-ion transference number increase together; this molecular tuning complements the network approach and supports higher-voltage operation [135]. A bio-derived route from Hadad et al. reaches the same objectives with a different lever: Figure 3d outlines the click-grafting pathway that attaches starch acetate to MXene quantum dots, setting up an electronically insulating but ionically conductive framework. Cell durability is then captured in Figure 3e, where full cells retain about 84.4, 87.9, and 90.1 percent of initial capacity after 1000 cycles for SA/MXene 10, 30, and 50 while maintaining Coulombic efficiency near 99–100 percent. Oxidative stability is mapped in Figure 3f, which shows an anodic limit around 5.5 V versus Li/Li^+^ for the optimized composition, and bulk transport is summarized in Figure 3g, where room-temperature conductivity lies in the millisiemens-per-centimeter regime with an Arrhenius trend on 1/T plots [123]. Seen together, these figures support one operational playbook: reduce crystallinity only until segmental motion improves, keep interfaces clean so impedance remains flat, and program MXene either by network formation or by surface grafting so continuous and ion-selective pathways convert transport gains into low-overpotential plating and long-life cells. The same logic explains use-case fit. The PEO/SN–MXene blend, which is introduced by Xu et al., is attractive for low-temperature endurance and high-rate operation but typically shows lower transference and requires tight crystallinity control. Ionic liquid-modified MXene extends the stability window and improves ion selectivity, though viscosity and cost must be managed. Continuous MXene scaffolds add abuse tolerance and interface quality but need processing control to avoid overdense networks that suppress conductivity [136].

MXene–polymer electrolytes stand out as the best-evidenced route after these five studies: polar terminations and light crystallinity suppression push conductivity into about 10^−4^ to 10^0^ S cm^−1^, raise the Li^+^ transference number, and support long, low-overpotential Li∥Li cycling, including near sub-zero. Networked or surface-grafted MXene co-optimizes transport, mechanics, and interface stability without heavy ceramic loading. The practical takeaway is simple: program MXene surface chemistry and mesoscale architecture to build continuous, ion-selective pathways, and verify with standardized transport and cell metrics.

#### 6.1.2. MXene–Polymer Separators in Lithium-Metal Batteries

**Figure 4:**
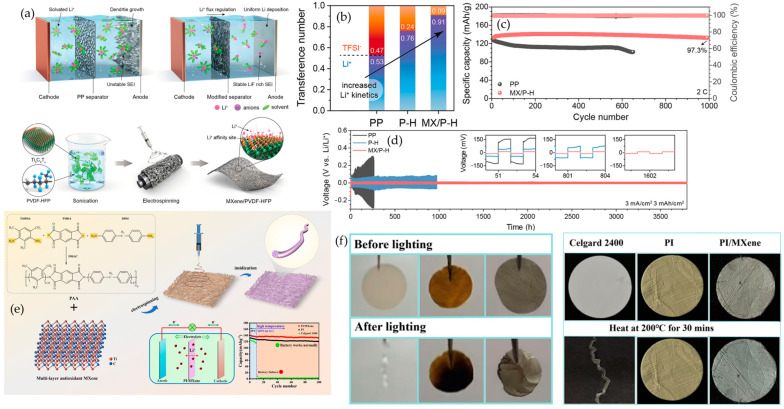
(**a**) Concept and fabrication of MXene/PVDF–HFP (sonication then electrospinning) showing a LiF-rich interphase that blocks electrons and conducts Li^+^; (**b**) Li^+^ and TFSI^−^ transference numbers for PP, P-H, and MX/P-H; (**c**) LFP∥MX/P-H∥Li cycling at 2 C for 1000 cycles; (**d**) Symmetric Li∥Li cycling at 3 mA cm^−2^ and 3 mAh cm^−2^ with stable plating and stripping; (**e**) Schematic and process of PI/MXene nanofiber separator obtained by electrospinning PAA and imidization to PI, forming an MXene-assisted porous network that preserves wetting and ion pathways; (**f**) Thermal photographs after heating at 200 °C for 30 min showing minimal shrinkage and flame tolerance for PI/MXene compared with PI and Celgard. Panels (**a**–**d**) reproduced with permission from Ref. [138] (Wiley-VCH, 2025); panels (**e**,**f**) reproduced with permission from Ref. [118] (Frontiers Media SA, 2025).

MXene–polymer architectures for lithium-metal batteries are best read as reactive, ion-selective, safety-relevant interfaces in which solvation chemistry, ion transport, and mechanical and thermal resilience are co-designed. The sequence in Figure 4a–f follows this logic. Xiang et al. begin with transport by converting MXene sheets into negatively charged nanochannels on electrospun PAN/PEI. The separator then favors cation motion: the Li^+^ transference number rises to about 0.67 relative to about 0.34 for polypropylene. Ionic conductivity increases to about 1.6 mS cm^−1^ and the contact angle falls to about 8°, and these gains carry into devices where Li∥NMC811 full cells retain about 91.2% after 200 cycles with 146.1 mAh g^−1^ at 5 C [137].

On this transport foundation, Kim et al. embed Ti_3_C_2_T_x_ into PVDF–HFP so that polar O, OH, and F terminations couple with the fluorinated matrix and catalyze TFSI^−^ decomposition into a LiF-rich interphase that blocks electrons while conducting Li^+^ (Figure 4a; the sonication-to-electrospinning route appears beneath). The interfacial regulation translates directly into ion selectivity and device life. The Li^+^ transference number rises stepwise from PP to P-H to MX/P-H, reaching about 0.91 for the MXene-engineered membrane while anion mobility is suppressed (Figure 4b). Li∥LFP full cells retain roughly 97.3% capacity after 1000 cycles at 2 C (Figure 4c). Li∥Li symmetric cells sustain about 3800 h of stable plating and stripping at 3 mA cm^−2^ and 3 mAh cm^−2^ (Figure 4d) [138]. Complementing this chemistry-led stability, Wu et al. provide the safety and electrolyte management pillar with PI/MXene nanofiber separators. The scheme and processing, which involve electrospinning followed by imidization from PAA to PI, yield a porous MXene-assisted network that preserves ion pathways and wetting (Figure 4e). Photographs after heating at 200 °C for 30 min show that PI/MXene resists flame and markedly limits thermal shrinkage compared with PI and Celgard (Figure 4f). High uptake and retention reported for this membrane align with the lower bulk resistance and higher conductivity measured against Celgard, so the same separator that tolerates heat also supports ion-friendly transport under lean electrolyte [118].

In summary, the three datasets are mutually reinforcing and they define a practical rulebook. First, raise Li^+^ selectivity and through-plane conductivity while maintaining a non-percolating MXene fraction so that the separator remains electronically insulating, as demonstrated by Xiang et al. Second, engineer LiF-rich, electron-blocking interphases using MXene in a fluorinated matrix so that uniform nucleation is sustained at 1 to 3 mA cm^−2^ and 1 to 3 mAh cm^−2^, as shown by Kim et al. Third, embed these functions in a scaffold that manages electrolyte and heat better than polyolefin baselines; the comparison with Celgard on resistance, conductivity, and operability up to 120 °C in Wu et al. shows a clear performance margin that is relevant for abuse tolerance and for high areal capacity designs. The complementary metrics make the case that MXene–polymer interfaces do not simply add tortuosity. They selectively carry Li^+^, they script the interfacial chemistry into a LiF-rich state, and they supply a thermally robust, electrolyte-philic backbone. The result is fast and uniform lithium plating, long symmetric-cell lifetimes, and strong full-cell figures under realistic current densities and loadings.

#### 6.1.3. MXene–Polymer-Programmed Solid Electrolytes Interphases (SEIs) in Lithium-Metal Batteries

A MXene–polymer solid electrolyte interphase (SEI) works by letting the polymer tune transport and chemistry on a Ti_3_C_2_T_x_ scaffold so lithium-ion pathways open while electrons remain confined, which softens nucleation and stabilizes cycling. In a β-phase poly(vinylidene fluoride) on Ti_3_C_2_T_x_ design, the interfacial charge becomes more surface-controlled, with the capacitive fraction about 61% at 0.2 mV s^−1^ compared with 37% on bare MXene; the lithium nucleation overpotential falls to roughly 30 mV, versus about 39 mV on MXene and about 88 mV on copper, and Coulombic efficiency averages around 97.3% for about 170 cycles in asymmetric half-cells. Under demanding operation, the interface remains robust, maintaining stable profiles for more than 300 h at 5 mA cm^−2^ and 5 mAh cm^−2^. Ex situ spectroscopy shows a LiF-dominated outer SEI together with Li_2_CO_3_ and Li_2_O, which aligns with the observed low overpotential, low hysteresis, and durable high-rate behavior [139].

Within the broader MXene–polymer family, covalent organic frameworks (COFs) can be viewed as crystalline porous polymers that provide ordered nanopores and densely distributed lithiophilic sites. A recent soft–rigid MXene/COF artificial SEI, in which COF spheres are grown on Ti_3_C_2_T_x_, uses the COF network as an ion-permeable polymer shell and the MXene as a mechanically robust electron backbone; the resulting MXene/COF@Li interlayer promotes fast, homogeneous Li^+^ transport, suppresses dendrite penetration, and significantly improves Li∥Li, Li∥LFP and Li∥NCM811 cycling compared with bare Li [140]. In a complementary design, a thin COF-1-modified MXene coating (COF–MXene–Li) employs boroxine-linked COF-1 as a lithiophilic polymer framework anchored on Ti_3_C_2_T_x_; this hybrid MXene–COF SEI increases Li nucleation density, stabilizes the interfacial impedance, and enables long-life Li∥NMC811 and pouch cells with higher capacity retention than uncoated Li [141].

Despite these gains, MXene–polymer SEI studies in lithium-metal batteries, including those extended to crystalline porous polymers such as MXene–COF hybrids, are still scarce. The design window is narrow. The interphase must remain electronically insulating while allowing fast Li^+^ transport, so MXene loading, connectivity, and dispersion have to stay below the percolation threshold and restacking must be controlled. For MXene–COF systems this is even stricter, because the COF must form a continuous, ion-permeable polymer network on Ti_3_C_2_T_x_ without creating short paths for electrons or blocking Li^+^ channels, and the covalent MXene–COF interface must survive long-term cycling. Variations in surface terminations, COF chemistry, and polymer compatibility further reduce reproducibility. It is also difficult to deposit thin, defect-free coatings on rough Li with scalable methods. Finally, practice-oriented tests such as high current density, high areal capacity, lean electrolyte, and long cycling quickly eliminate weak designs, so truly robust MXene–polymer and MXene–COF SEI examples remain rare.

#### 6.1.4. MXene–Polymer Electrodes (Anode and Cathode) in LMBs

A Li-metal cell rewards electrodes that decide where charge begins and how it flows through the thickness; when current is homogenized and interfaces are moderated, the cell spends less of its impedance budget fighting morphology and more delivering power. Viewed through this lens, a single design language emerges: a thin, electronically continuous MXene skeleton kept below through-thickness percolation for wiring and current smoothing, paired with a polymer that performs interfacial work such as ion-selective moderation, adhesion, and elastic buffering.

On the lithium side, two complementary hosts control where lithium nucleates and how it grows without relying on figure references. Shi et al. graft a P4VP brush onto Ti_3_C_2_ and chelate Ag^+^ so that 4–15 nm Ag seeds define uniform nucleation sites; the result is a millivolt-scale nucleation overpotential (3 mV), Coulombic efficiency consistently above 98% for 200 cycles, and stable plating/stripping over hundreds of hours, with LFP full cells sustaining 102.8 mAh g^−1^ at 5 C and retaining 94% after 150 cycles [142]. Wang et al. arrive at the same endpoint through topology by stitching Ti_3_C_2_ lamellae with a trace of cellulose nanofiber into a 25 μm self-supporting paper that preserves lateral electronic continuity and opens vertical ion corridors; this scaffold maintains low polarization (60 mV) at 1 mA cm^−2^ with 2 mAh cm^−2^, keeps Coulombic efficiency near 98–99% for 200 cycles, and extends Li∥Li lifetimes to the order of 10^3^ hours, indicating homogenized current without through-thickness shortcuts [143].

**Figure 5:**
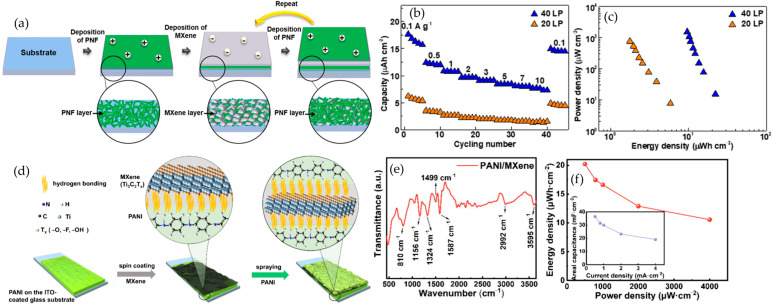
(**a**) Layer-by-layer assembly schematic and film architecture. (**b**) Rate response for films with varying layer pairs. (**c**) Areal Ragone plot showing thickness scaling. (**d**) Sandwich fabrication and hydrogen bonding interactions. (**e**) Raman spectra indicating polymer–MXene coupling. (**f**) Device-level Ragone and cycling stability. Panels (**a**–**c**) reproduced with permission from Ref. [144] (American Chemical Society, 2019); panels (**d**–**f**) reproduced with permission from Ref. [145] (American Chemical Society, 2024).

On the cathode side, Yun et al. alternate positively charged polyaniline nanofibers with negatively charged Ti_3_C_2_T_x_ so each bilayer remains electrochemically addressable. The assembly concept and architecture appear in Figure 5a. The rate response in Figure 5b quantifies thickness-accessible behavior, where the 40-pair film decreases from about 17.6 to 7.3 μAh cm^−2^ as discharge current increases and then recovers to roughly 15 μAh cm^−2^ when returned to 0.1 A g^−1^; baseline capacities also scale with layer pairs at about 2.3, 6.2, 10.2, and 16.4 μAh cm^−2^ for 10, 20, 30, and 40 pairs, respectively. The areal Ragone in Figure 5c confirms clean thickness scaling for 2 μm films, reaching about 17.6 μAh cm^−2^ and 22.1 μWh cm^−2^ while delivering around 1.5 mW cm^−2^ [144]. In a complementary design, Lu et al. sandwich a thin MXene layer between polyaniline skins. Figure 5d outlines the fabrication and the hydrogen bonding interactions that widen the MXene (002) spacing from 14.88 Å to 15.34 Å and lower charge-transfer resistance [145]. Figure 5e then resolves the bonding at the molecular level, where characteristic Raman bands of polyaniline such as quinoid C=N and benzenoid C–N stretching appear alongside MXene-related Ti–C and Ti–O modes, and the relative intensities with slight shifts indicate strong interfacial coupling rather than a simple physical mixture, which is consistent with widened galleries and preserved conjugation across the stack. Finally, Figure 5f translates these interfacial gains into device behavior, since the composite’s Ragone traces occupy a higher energy-at-power envelope than polyaniline-only or MXene-only controls, the energy–power trade-off remains monotonic as current increases with minimal hysteresis on returning to a low rate, and the microdevice retains its electrochemical and optical response over approximately 10^3^ cycles, which shows that the architecture converts structural advantages into durable energy and power at the film scale.

Taken together, these studies suggest a practical design framework: use chemistry on MXene to decide where lithium begins and how it grows, use topology to keep ion corridors open and current uniform, and on the positive electrode keep redox polymer domains addressable while MXene galleries stay wet and conductive. The common outcome is even Li^+^ flux, low polarization, and durable cycling at the device level, seen as long-life symmetric cells and high-rate, high-retention LFP full cells on the anode, and thickness-scalable energy and power in the PANI∥MXene films on the cathode.

**Figure 6:**
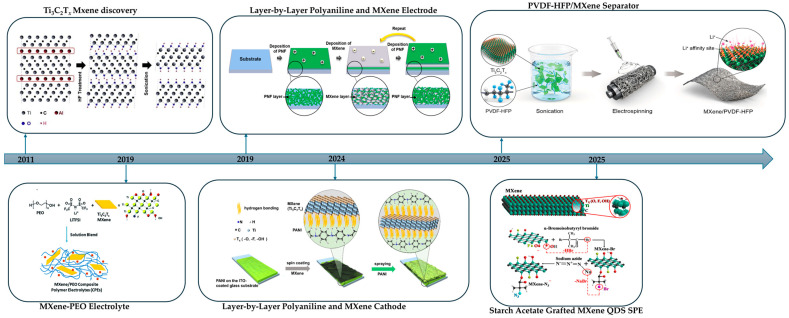
Milestones of MXene–polymer strategies for lithium-metal batteries across electrolyte, separator/SEI, and cathode. Reproduced with permission from Ref. [10] (Wiley-VCH, 2011); Ref. [86] (Royal Society of Chemistry, 2019); Ref. [123] (Wiley-VCH, 2025); Ref. [138] (Wiley-VCH, 2025); Ref. [144] (American Chemical Society, 2019); Ref. [145] (American Chemical Society, 2024).

In summary, recent studies indicate that MXene–polymer hybrids are not just fillers but a coherent design platform across electrolytes, separators, SEI, and electrodes when MXene surface chemistry, polymer interactions, and ion transport architecture are optimized together. Matching MXene terminations to compatible polymers reduces crystallinity, improves salt dissociation, and tunes solvation to deliver stable Li^+^ flux, while grafted or coated polymer layers on MXene preserve ionic conduction and block electronic leakage, enabling smoother lithium plating and more robust SEI. In separators, the 2D morphology and surface charge of MXene enhance cation selectivity and homogenize current distribution, and in ceramic–polymer systems the sheets act as bridges that lower interfacial impedance without sacrificing toughness. Ordered MXene papers or interlayers in electrodes create thickness-accessible pathways for both ions and electrons. The practical path forward is to standardize testing under application-relevant constraints, favor simple scalable fabrication such as casting, electrospinning, and roll-to-roll layering, and control oxidation and restacking from the outset via greener syntheses. With these elements aligned, MXene–polymer systems can credibly support room-temperature solid-state lithium-metal batteries with stable, reproducible performance. However, dedicated studies on MXene–polymer-engineered SEI remain limited; key gaps include a consistent mechanistic map from MXene terminations to SEI chemistry/morphology under practical cycling and standardized protocols to compare SEI durability and failure modes. These ideas are summarized in Figure 6, which maps milestones from MXene discovery to polymer electrolytes, solvation-regulated separators and SEI, and layer-by-layer electrodes.

### 6.2. Lithium–Sulfur Batteries

Lithium–sulfur batteries face intertwined transport and interfacial challenges: elemental sulfur and lithium sulfide are insulating, soluble polysulfides migrate and react parasitically, the conversion between sulfur and lithium sulfide is kinetically sluggish, and the cathode undergoes significant volume change during cycling. MXene–polymer hybrids offer a coherent strategy to address these issues by pairing MXene’s electronic conductivity and polar, adsorptive terminations with processable polymer frameworks that regulate porosity, ion transport, and mechanical integrity [146,147,148]. In separator engineering, thin MXene–polymer coatings or porous skins increase electrolyte wettability, introduce Li^+^-selective pathways, and provide targeted polysulfide affinity while keeping the electronic network below through-thickness percolation so that shuttle is suppressed without risking shorting [146,148]. As cathode hosts, MXene–polymer matrices establish bicontinuous electron and ion pathways, immobilize polysulfides through surface interactions, and buffer strain, which supports high sulfur loading and lean electrolyte operation with stabilized redox fronts [146,147]. Within electrolytes, MXene–polymer composites, including gel and solid polymer systems, can raise room-temperature ionic conductivity, increase the lithium-ion transference number by anchoring anions, and steer interfacial chemistry toward faster, more uniform conversion between sulfur and lithium sulfide [146,147,148]. The following sections synthesize evidence across separators, cathode hosts, and electrolytes, emphasizing structure–property–performance links and practical metrics such as areal capacity, rate capability, electrolyte-to-sulfur ratio, and long-term retention.

#### 6.2.1. MXene–Polymer Separators in Lithium–Sulfur Batteries

**Figure 7:**
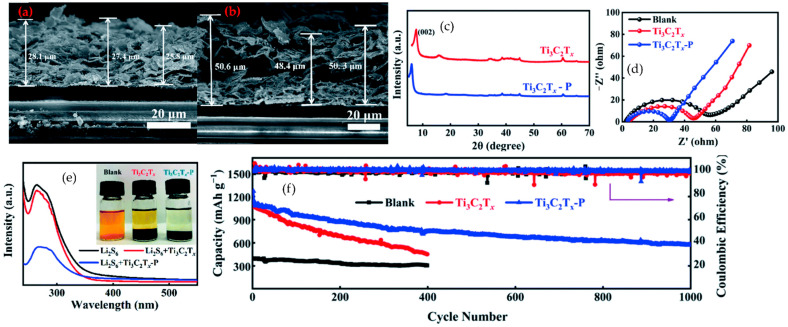
(**a**) Cross-sectional SEM of Ti_3_C_2_T_x_/PP separator. (**b**) Cross-sectional SEM of Ti_3_C_2_T_x_–P/PP separator. (**c**) XRD patterns showing the (002) reflection shifting to lower 2θ upon PEDOT:PSS intercalation. (**d**) EIS spectra comparing cells with PP, Ti_3_C_2_T_x_/PP, and Ti_3_C_2_T_x_–P/PP separators. (**e**) UV–vis spectra and vial photographs of electrolytes collected from the permeate side of H-cell devices after Li_2_S_6_ permeation tests. (**f**) Long-term cycling and Coulombic efficiency for cells using a Ti_3_C_2_T_x_–P/PP separator. Panels (**a**–**f**) reproduced with permission from Ref. [150] (Royal Society of Chemistry, 2020).

Co-designed MXene–polymer separators work because a single ultrathin interface is engineered to conduct electrons, select Li^+^, and regulate polysulfides, and not because the separator soaks up LiPS by brute force. In Wang et al., a laminar Ti_3_C_2_T_x_–Nafion skin on PP makes this logic explicit: sulfonate groups impose Donnan exclusion, while hydrated channels inside the MXene galleries guide Li^+^. At a low loading of only 0.2 mg cm^−2^, H-cell photographs already show markedly slower LiPS permeation. The same film sustains high-rate cycling for about 1000 cycles at 1 C and outperforms dual-layer MXene@Nafion and GO–Nafion at a similar mass, indicating that intimate sheet–polymer mixing and carefully calibrated thickness, rather than extra loading, govern transport and durability [149]. Pushing this concept further, Li et al. apply the same principle by intercalating PEDOT:PSS into Ti_3_C_2_T_x_ so that the electron pathway remains continuous yet sub-percolating, while the PSS polyanion contributes ion selectivity and LiPS rejection. In the PEDOT:PSS figure here, Figure 7a,b shows the cross-sectional SEM where the coating thickens from roughly 25–28 μm for Ti_3_C_2_T_x_/PP to about 48–56 μm for Ti_3_C_2_T_x_–P/PP, and Figure 7c records the (002) XRD peak shifting to lower 2θ which evidences widened galleries and improved electrolyte uptake. Figure 7d shows a smaller Nyquist semicircle that reflects a drop in charge-transfer resistance and faster interfacial kinetics, while Figure 7e shows the UV–vis vial and spectra where the Ti_3_C_2_T_x_–P system suppresses Li_2_S_6_ absorbance. Figure 7f translates these mesoscale changes into durable full-cell behavior with high Coulombic efficiency, and numerically the study reports an initial capacity near 1241 mAh g^−1^ at 0.2 C and an ultralow capacity decay of about 0.030% per cycle to 1000 cycles at 0.5 C, which ties structural control to kinetic stability rather than listing capacity as an isolated metric [150]. Extending the co-design toward safety, Li Y. et al. co-assemble a DNA–CNT/MXene network so that phosphate backbones and polar MXene sites curb Li_2_S_8_ crossover while CNT and MXene preserve percolation. H-cell UV–vis confirms attenuated Li_2_S_8_ transport, and the charge-transfer resistance falls from about 82.8 Ω to about 13.9 Ω. The cell still retains about 79% capacity after 200 cycles at 75 °C with high Coulombic efficiency, showing that the same mixed-conduction and permselective chemistry can be maintained under thermal stress [151]. Taken together, these results show that ultrathin, mass-calibrated MXene–polymer interfaces can merge conduction with ion selectivity and LiPS anchoring. In practice, this converts transport control into faster interfacial kinetics, longer cycle life, and better high-temperature resilience.

In summary, these three experiments support one another across both mechanisms and metrics. The polymer component (Nafion, PEDOT:PSS, or DNA) sets ion selectivity, dispersion, and wetting. The MXene lamellae provide fast electron pathways and strong LiPS binding, while the one-sided, lean coating geometry keeps ionic access open. Under realistic constraints that matter for translation—for example, electrolyte-to-sulfur ratio near or below 5 μL mg^−1^, sulfur loading above ≈5 mg cm^−2^ with areal capacity near or above 4 mAh cm^−2^, controlled negative-to-positive capacity ratio, and sustained operation at 1–3 C with rate recovery—this playbook explains why MXene–polymer separators consistently outperform polymer-only or inorganic-only analogs. They deliver advantages under practical conditions rather than only as isolated record cells.

#### 6.2.2. MXene–Polymer Electrolytes in Lithium–Sulfur Batteries

**Figure 8:**
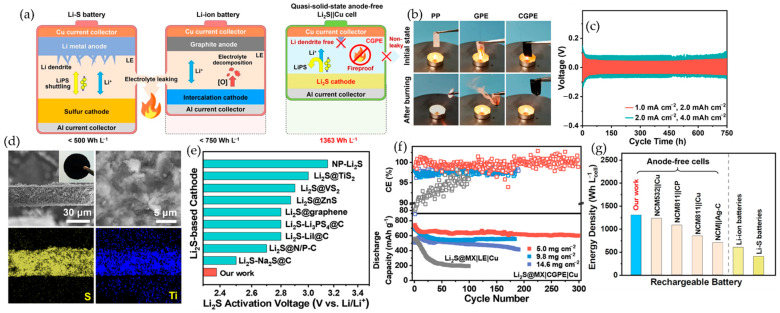
(**a**) Schematic comparison of Li–S, Li-ion, and a quasi-solid anode-free Li_2_S∥Cu configuration using a MXene-doped fluorinated CGPE. (**b**) Flame-resistance test of PP, GPE, and CGPE membranes. (**c**) Long-term Li∥Li symmetric cycling with CGPE. (**d**) Cross-sectional morphology and elemental mapping of a Li_2_S@MXene cathode. (**e**) Comparison of Li_2_S activation voltages across hosts. (**f**) Cycling performance and Coulombic efficiency at increasing areal loadings with Li_2_S@MXene|CGPE cells. (**g**) Volumetric energy density benchmark for anode-free pouch cells. Panels (**a**–**g**) reproduced with permission from Ref. [152] (Springer Nature, 2022).

Electrolytes ultimately decide whether sulfur redox remains local to the cathode or diffuses across the cell, because composition fixes polysulfide solubility and shuttle [7]. Figure 8a frames this contrast by placing conventional Li–S and Li-ion next to a quasi-solid-state anode-free Li_2_S∥Cu architecture, where a Li_2_S@MXene cathode is paired with a non-flammable, MXene-doped fluorinated composite gel polymer electrolyte so that transport, interphase chemistry, and safety are regulated within one medium. In Liu et al., this CGPE delivers millisiemens-class ionic transport while remaining electronically insulating, and the figure makes the consequences visible: the flame test in Figure 8b keeps the membrane intact while PP and a liquid-plasticized GPE deform, and the symmetric Li∥Li trace in Figure 8c stays essentially flat for hundreds of hours under 1.0–2.0 mA cm^−2^ and 2.0–4.0 mAh cm^−2^ windows. The microstructure and chemistry on the cathode side support the same story. Figure 8d shows a compact Li_2_S@MXene framework with sulfur and titanium co-localized in cross-section and elemental maps, which improves interfacial contact and shortens Li^+^ diffusion paths; Figure 8e benchmarks the lowered Li_2_S activation voltage against the literature composites, linking MXene contact and polar surfaces to easier Li_2_S activation; Figure 8f preserves high Coulombic efficiency and stable capacities as areal loading rises from 5.0 to 9.8 and 14.6 mg cm^−2^; and Figure 8g places the pouch-cell energy density near 1.3 kWh L_cell^−1^ among anode-free references, aligning safety and performance rather than trading one for the other [152]. To isolate the role of MXene, Li et al. provide a strong polymer-only baseline: a PEO–PGA composite polymer electrolyte reaches σ = 4.9 × 10^−4^ S cm^−1^ and tLi^+^ = 0.53 at 50 °C and sustains Li∥Li cycling beyond 2800 h at 0.2 mA cm^−2^, which confirms that competent polymer transport and interfacial stability are achievable without MXene [153].

Against that baseline, Liu et al.’s CGPE does not simply exchange conductivity for safety. It matches room-temperature transport, adds stronger shuttle control, and remains thermally resilient, so the gains appear together in kinetics, durability, and abuse tolerance [128]. Beyond gels, PEO-based solid electrolytes that integrate MXene follow the same pattern: Arrhenius plots shift upward relative to neat PEO/LiTFSI and transference numbers rise toward 0.35–0.40, and Li–S cells retain higher capacity at 0.5 C and 60 °C than the polymer alone; DFT and XPS indicate that MXene terminations provide polar binding sites that suppress polysulfide activity in the membrane, so improved transport does not come at the expense of shuttle control [154].

To summarize, MXene only adds value in electrolytes when it is used to engineer the medium itself, turning it into a selective, ion-wiring yet electronically insulating matrix rather than a passive carrier. The playbook is clear. MXene loading should stay below electronic percolation so that it guides ions without creating electronic shortcuts. The polymer host must match the operating window, and the MXene loading and membrane thickness should be tuned so that transport, interphase quality, and thermal robustness improve together instead of trading off. Mechanistically, polar terminations regulate anions and polysulfides, stiff lamellae smooth ionic flux and dissipate local heat, and the polymer preserves insulation while providing wetting, adhesion, and mechanical damping. Methodologically, verify the full triad by coupling membrane-level transport with symmetric-cell stability and full-cell behavior at practical loading, and report safety alongside kinetics. Applied this way, MXene turns the electrolyte into an active regulator of transport, interphase chemistry, and safety, aligning all the key performance levers in the same direction.

#### 6.2.3. MXene–Polymer Cathodes in Lithium–Sulfur Batteries

**Figure 9:**
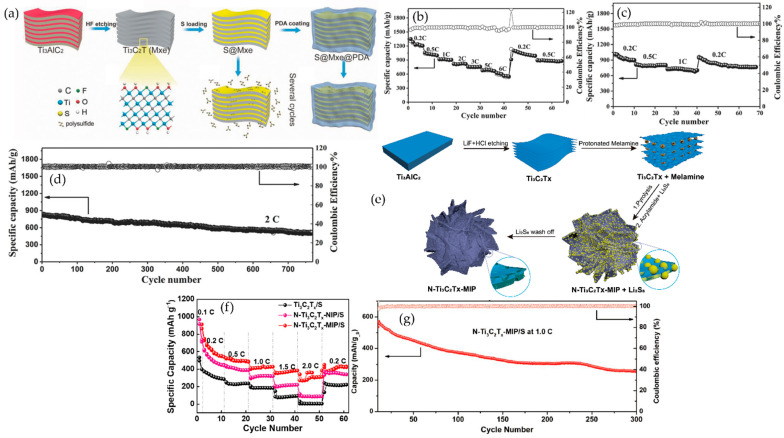
(**a**) Schematic of MXene-based sulfur host preparation and thin polymer coating for dual confinement. (**b**) Stepwise rate capability of the coated S@MXene cathode. (**c**) Cycling stability and Coulombic efficiency of the coated cathode at practical rates. (**d**) Long-term cycling of a conjugated-microporous-polymer-grafted Ti_3_C_2_T_x_/S host at high rate. (**e**) Concept of a Li_2_S_8_-imprinted polyacrylamide layer on N-doped Ti_3_C_2_T_x_ with specific recognition of polysulfides. (**f**) Rate comparison among Ti_3_C_2_T_x_/S, N-Ti_3_C_2_T_x_–NIP/S, and N-Ti_3_C_2_T_x_–MIP/S. (**g**) Extended cycling and Coulombic efficiency at 1 C for the N-Ti_3_C_2_T_x_–MIP/S cathode. Panels (**a**–**c**) reproduced with permission from Ref. [155] (Wiley-VCH, 2018); panels (**e**–**g**) from Ref. [156] (Elsevier, 2025).

Cathode performance in Li–S cells improves most when MXene surfaces and polymer chemistry are co-designed so adsorption selectivity, catalytic conversion, ion transport, and mechanical integrity act in concert. Figure 9a shows the dual confinement scheme of Yao et al., where S is intercalated in Ti_3_C_2_T_x_ and a nanometric polydopamine skin supplies an ion-permeable, adhesive sheath that works with Ti–S bonding inside the galleries. Figure 9b preserves clean discharge plateaus under stepwise current, indicating shuttle suppression without throttling kinetics; Figure 9c keeps the step changes stable with high Coulombic efficiency, which signals that conversion stays local to the scaffold; Figure 9d extends this to long-term operation at practical rates and at high sulfur loading, including the demanding case of about 4.4 mg cm^−2^ and rate tests up to 6 C, so confinement and contact scale with device relevance [155]. Figure 9e then turns selectivity into recognition in Yan et al., where a Li_2_S_8_-imprinted polyacrylamide on N-doped Ti_3_C_2_T_x_ presents template-matched pockets that guide Li_2_S_x_ toward catalytic MXene sites. Figure 9f compares a sharper stepwise rate profile with non-imprinted and bare MXene controls, and Figure 9g converts that chemistry into durable 1 C cycling with sustained Coulombic efficiency, showing that recognition, catalysis, and conduction are co-located rather than traded off [156]. Complementing these image-based cases, Cao et al. graft a conjugated microporous polymer directly onto Ti_3_C_2_T_x_ so that binding sites sit on a continuous electron backbone, and the cathode fades by only about 0.025% per cycle over 1000 cycles at 2 C, which is the signature of genuine on-scaffold conversion rather than temporary trapping [157].

Read together, these results support a single working rule for MXene–polymer Li–S cathodes: place specific chemistry exactly where ions and electrons meet, so that confinement, recognition, and catalysis are embedded in a conductive and mechanically coherent host, and the gains in rate response and long-life cycling emerge without numerical excess.

In sum, the evidence points to a simple working rule for Li–S cathodes: design the MXene surface chemistry and the polymer functionality as one integrated interface so that electrons and ions move together and polysulfides are captured and converted exactly where they arrive. Conjugated networks grafted to Ti_3_C_2_T_x_ supply chemisorption along a continuous electronic backbone. Nanometric polymer skins add permeable confinement that preserves intimate contact without throttling transport. Molecularly imprinted layers introduce recognition that delivers the appropriate Li_2_S_x_ species to catalytic sites. The result is cooperation rather than trade-off: cleaner voltage plateaus at high rates, stable capacities at practical sulfur loadings, and compact post-cycling textures with good Coulombic efficiency. In effect, the cathode stops being a passive storage scaffold and becomes a programmed reaction environment in which selective adsorption, catalytic conversion, ion conduction, and mechanical coherence reinforce one another over long service.

**Figure 10:**
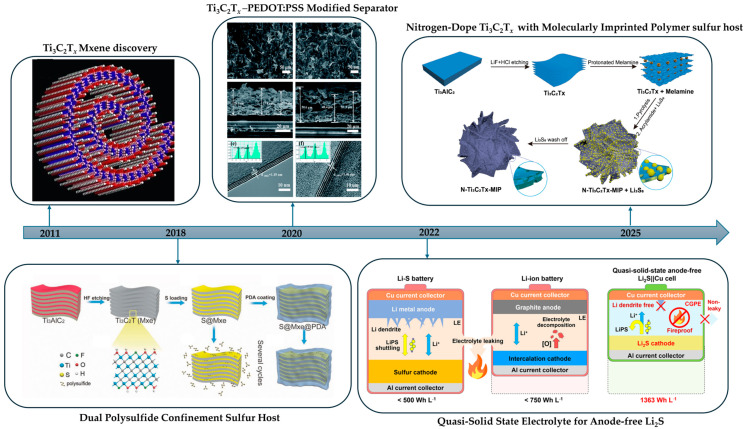
Year-by-year milestones of MXene–polymer strategies for Li–S batteries across separator, electrolyte, and cathode. Reproduced with permission from Ref. [10] (Wiley-VCH, 2011); Ref. [150] (Royal Society of Chemistry, 2020); Ref. [152] (Springer Nature, 2022); Ref. [155] (Wiley-VCH, 2018); Ref. [156] (Elsevier, 2025).

In brief, MXene–polymer strategies in Li–S batteries reframe separators, electrolytes, and sulfur hosts as active, ion-selective, and catalytic networks. Separators use MXene–polymer chemistry to bind and regulate polysulfides while smoothing Li^+^ flux and improving thermal robustness. Polymer electrolytes leverage MXene to raise Li^+^ conductivity, temper crystallinity, and add selective adsorption that stabilizes transport. Cathode hosts couple polar adsorption with catalytic conversion while limiting electronic leakage so that conversion remains efficient at practical sulfur loading. Taken together, these advances point to an integrated design that keeps flux, selectivity, and electronic blocking consistent across the separator, electrolyte, and cathode. Two clear gaps remain for Li–S: despite their strong performance, MXene–polymer electrolytes have limited systematic work on non-flammable, flame-retardant formulations and safety under abuse conditions, and there are essentially no dedicated studies on MXene–polymer-engineered solid electrolyte interphases for the lithium anode. The next step is to design Li-facing MXene–polymer interphases and, in parallel, develop fire-safe electrolyte architectures, then validate both under lean electrolyte, high areal loading, and long-duration cycling so interphase stability and safety co-evolve with cell-level transport control. Figure 10 summarizes these ideas as a year-by-year milestone timeline across the separator, electrolyte, and cathode.

In summary, HF-etched Ti_3_C_2_T_x_ offers mixed F, O, and OH terminations that improve wetting and often seed a LiF-rich, electron-blocking interphase at the lithium. This interphase stabilizes plating and stripping in lithium-metal cells and strengthens polysulfide control in lithium–sulfur designs. In polymer-based architectures, MXene is embedded in an electronically insulating matrix that supplies ion selectivity, adhesion, and elastic buffering. Electrodes perform best when a laterally continuous yet sub-percolating MXene framework is co-designed with a binder or ionomer so that ionic pathways are preserved and current is smoothed. Gel electrolytes and modified separators benefit from polar MXene surfaces and widened galleries, which lower interfacial resistance and support fast Li^+^ transport at practical loading. SEI-oriented coatings formed or cured on MXene guide ion-selective pathways and sustain high-rate cycling. Against this HF-anchored baseline, fluoride-free MXenes bring O or OH or Cl or S terminations and shift optimization toward electrolyte and additive programming, as well as matrix formulation and curing control, while preserving the same architectural rules. Practical demonstrations are growing but remain limited, especially for lithium-metal and lithium–sulfur batteries, so the following discussion shows how these greener routes translate the same MXene–polymer design into real devices.

## 7. Green-Synthesized MXene–Polymer Composites for Lithium Batteries

HF-based routes made MXenes accessible and showcased their high conductivity and polar surfaces, but they also created safety and environmental burdens, left residual fluoride that perturbs interfacial chemistry, and produced variable terminations that complicate dispersion and bonding in polymers. In response, fluoride-free syntheses such as Lewis-acid molten-salt etching, electrochemical etching or delamination, and halogen- or alkali-assisted hydrothermal methods now yield MXenes with cleaner O/OH/Cl/S-rich terminations and more predictable compatibility with polymer matrices. This section focuses on how these fluoride-free MXene–polymer composites are used in two battery classes: lithium-metal batteries, and lithium–sulfur batteries.

### 7.1. Lithium-Metal Batteries

Recently, only two studies have examined green MXene–polymer systems specifically engineered as SEIs grown directly on lithium, both using fluoride-free routes and demonstrating SEI-focused advantages. Across both studies, the fluoride-free MXene–polymer SEI follows one design idea: synthesize Cl/O-terminated Ti_3_C_2_T_x_ by a Lewis-acid molten-salt route, disperse the sheets in a reactive monomer, and cure directly on Li so that the interphase is firmly bound, mechanically reinforced, ion-selective, and still electronically non-percolating [97,98]. In the first study (Huang T.), Li∥SSE∥Li cells show low, flat polarization for about 1000 h at 0.20 mA cm^−2^, while AFM maps indicate a modulus increase from roughly 2–3.3 GPa to 20–35 GPa that aligns with smoother plating; LFP full cells maintain about 91% capacity after 900 cycles, supported by a Li^+^ transference number of 0.512 and an operating optimum near 5 wt% MXene that avoids both under-modification and aggregation [97]. In the second study (Huang Z.) using an MMA matrix, symmetric cells exhibit small, steady polarization across the tested current densities, LFP full cells deliver around 106.6 mAh g^−1^ after 1000 cycles with roughly 80% retention, the impedance reaches a minimum near 5 wt% MXene, and the cured MM-SEI attains mS-level ionic conductivity with a visibly smoother plating front than the polymer-only control [98].

Both datasets point to the same mechanism. Sheet-reinforced polymer interphases redistribute current, increase interfacial stiffness, and generate LiF-enriched but cleaner chemistries even though the MXene itself is fluoride-free. In these systems, LiF arises predominantly from electrolyte-salt decomposition rather than MXene-borne fluorides. In practice, keeping the MXene loading near 5 wt% and curing the polymer uniformly in situ preserves an in-plane continuous network for field smoothing and stress sharing while avoiding through-thickness shortcuts. This architecture translates into durable, low-polarization Li‖Li cycling and long-life LFP‖Li performance. From a performance standpoint, these fluoride-free SEIs already operate in a similar window to the best HF-derived Ti_3_C_2_T_x_/β-PVDF artificial SEIs. This suggests that, once the composite architecture and filler loading are optimized, the synthesis route mainly tunes interphase composition and fluorine content rather than fundamentally limiting Li-metal cycling stability.

At the same time, truly fluoride-free MXene–polymer interphases in lithium-metal batteries remain scarce, with the two molten-salt Ti_3_C_2_T_x_ studies standing as the clearest implementations, because fluoride-free syntheses are less mature and termination-sensitive, polymer integration on reactive lithium requires stable dispersion and controlled curing while avoiding electronic percolation, mechanistic attribution is harder when LiF forms in operando from the salt, and the field still leans on HF-etched precedents; taken together, the two studies demonstrate viability and synergy while highlighting an underexplored and promising direction for targeted development.

### 7.2. Lithium–Sulfur Batteries Li-S Batteries

To date, there is no dedicated experimental study that systematically couples a green or fluoride-free MXene with a polymer matrix specifically for lithium–sulfur batteries. Existing green MXene work that touches Li–S mostly uses the MXene itself as a sulfur host, interlayer, or catalytic coating rather than forming a true MXene–polymer composite; for example, fluorine-free Ti_3_C_2_ obtained by a photo-Fenton route and deployed directly in Li–S cells [55], Ti_3_C_2_T_x_ prepared via an environment-friendly molten-salt etch and evaluated as a sulfur host [158], and a dry molten-salt synthesis that yields sulfur-terminated Ti_3_C_2_ designed as a multifunctional Li–S catalyst or separator coating [159]. Consequently, a direct, metric-by-metric comparison between HF-derived and fluoride-free MXene–polymer composites, as can be carried out for Li-metal cells, is not yet possible for Li–S systems. The quantitative benchmarks in this review therefore refer to HF-etched MXene–polymer separators and gel electrolytes, whereas existing green MXene studies in Li–S employ fluoride-free Ti_3_C_2_T_x_ mainly as sulfur hosts, interlayers, or catalytic coatings without a polymer matrix.

The absence likely stems from chemistry and processing constraints unique to fluoride-free routes, where termination sets and surface energies vary across photo-Fenton, electrochemical, and Lewis-acid molten-salt methods, which complicates delamination, dispersion stability, oxidation control, and polymer co-processing; batch variability and residual salts or halides can further disturb ink rheology, curing, and the design of sub-percolating electronic networks inside polymer matrices [41,52]. At the same time, Li–S composite studies that integrate polymers have largely relied on HF-etched MXenes with stable surface chemistry and predictable processing windows, so the community has prioritized validating green MXene-alone efficacy before moving to polymer hybrids [160].

A reasonable outlook is that, as termination control, delamination quality, and impurity removal improve for green MXenes, in situ ionomer or gel polymer strategies that keep electron transport below percolation while leveraging strong polysulfide chemisorption on fluoride-free surfaces will enable the first true green MXene–polymer Li–S composites. These systems should then be benchmarked head-to-head against existing photo-Fenton, molten-salt, and sulfur-terminated exemplars.

Overall, HF-etched and fluoride-free MXenes map a complementary design space across both LMB and Li-S systems. HF-etched Ti_3_C_2_T_x_ brings mixed -F, -O, and -OH terminations that improve wetting and tend to seed a LiF-rich, electron-blocking interphase at the lithium. This gives predictable coupling with polymer matrices, smooths current in sub-percolating MXene frameworks, and supports fast transport through widened galleries. Lithium-metal cells benefit from lower nucleation overpotential and stable plating and stripping. Lithium–sulfur configurations gain stronger polysulfide control at practical loading. Fluoride-free routes deliver -O or -OH or -Cl or -S terminations and avoid inherited F, so interphase chemistry is driven mainly by electrolyte and additive programming together with curing control of the matrix. Processing is greener, yet data remain thinner for matrix-integrated devices, especially in Li–S. Across both routes the architectural rules stay the same. Keep the MXene network laterally continuous but below through-thickness percolation, keep the host electronically insulating, and use the interphase to direct ion-selective pathways. In practice, prefer HF-etched MXenes when a LiF-inclined interphase and high coupling reliability are desired from the outset. Prefer fluoride-free MXenes when greener handling and tailored interphases are prioritized, with electrolyte design providing the leverage to reach comparable durability.

## 8. Conclusions and Outlook for Green MXene–Polymer Composites in Li-Metal (LMB) and Li–S Batteries

### 8.1. Conlusions

MXene–polymer composites have transitioned from simple additives into programmable components for Li-metal and Li–S batteries. The synthesis route fixes surface terminations and interlayer spacing, while careful matching to polymer chemistry and processing governs wetting, ion transport, interphase chemistry, and membrane mechanics. HF-derived F-rich MXenes and fluoride-free green MXenes operate as complementary levers: the former can assist LiF-containing interphases, while the latter provide cleaner O-, OH-, Cl-, or S-rich surfaces that disperse predictably and align with safety and sustainability. In practice, the composite toolkit consolidates into five methods: solution blending and film casting, in situ polymerization, surface grafting, layer-by-layer assembly, and electrospinning. Solution blending and casting produce uniform matrices while keeping MXene below electronic percolation. In situ polymerization cures MXene–polymer networks either in bulk films or directly on lithium to form compact MXene–polymer SEI. Surface grafting builds covalent or ionic bridges for durable dispersion and stress transfer. Layer-by-layer assembly programs gradient or Janus architectures with controlled MXene loading. Electrospinning creates high-surface ion corridors with tunable porosity and mechanics. These routes enable four recurring pillars at device level: termination-guided wetting and interphase formation at lithium, widened and organized galleries that facilitate two-dimensional ion conduction at fixed thickness, plate-like reinforcement that maintains electronic insulation, and bifunctional polysulfide management that couples selective adsorption with catalytic conversion. Moving from demonstration to deployment now depends on disciplined benchmarking and transparent reporting of limitations. At minimum, studies should co-report a core set of parameters: ionic conductivity at 25 °C, lithium-ion transference number, storage modulus, and critical current density. They should also include termination fractions with transparent XPS fitting, interlayer spacing statistics, humidity-controlled contact angles, and flammability metrics, together with electrolyte-to-sulfur ratio, negative-to-positive capacity ratio, stack pressure, and operating temperature. With these reporting standards in place and with scalable implementations of the five methods above, green MXene–polymer composites become promising candidates for auditable, roll-to-roll manufacturing in lean electrolyte Li-metal cells and high-loading Li–S batteries.

### 8.2. Challenges and Practical Solutions

The field still faces cross-cutting hurdles alongside route-specific issues, so each problem needs a practical remedy, clear metrics, and a path to scale. For green routes, the main bottlenecks are termination control and oxidation or restacking in water or alcohol. Practical mitigation includes low-temperature anneals, brief plasma or UV treatments, ligand exchange, antioxidants, gentle vacuum drying and pillaring, followed by transparent XPS analysis, and interlayer spacing statistics to verify the surface chemistry. Several fluoride-free syntheses are also operationally harsh because they rely on high temperatures, aggressive salts, or specialized reactors such as hydrothermal autoclaves and Lewis-acid molten-salt furnaces. These conditions increase energy and capital cost and make it harder to maintain uniform surface terminations and morphology across large batches, so closed-loop handling, corrosion-resistant hardware, and in-line quality control are essential.

Scalability differs by synthesis. HF etching is throughput-proven but carries safety, efficient neutralization, and fluorine-residue cleanup burdens that complicate polymer compatibility and increase operating cost. Lewis-acid molten-salt etching yields fluoride-free, Cl/O-rich surfaces, but it is heat- and salt-intensive and therefore benefits from closed-loop salt recovery, corrosion-resistant hardware, and in-line impurity monitoring. Electrochemical etching is modular and stackable, yet it requires uniform current distribution, careful electrolyte management, and practical schemes for electrode reuse. Halogen and alkali hydrothermal variants are accessible, but they are often batch-limited and need reactor scale-out and impurity analytics.

Across the five composite fabrication methods, namely solution blending and film casting, in situ polymerization, surface grafting, layer-by-layer assembly and electrospinning, ion transport, and mechanical properties are mainly determined by MXene dispersion and interfacial bonding. In practice, formulations should match solvent and polymer polarity to the MXene terminations and contact angle measurements at controlled humidity can confirm wetting and adhesion. Chemical or ionic coupling agents can then help to fix a uniform network while keeping the MXene content below the electronic percolation threshold. For lithium-metal anodes, compact fluorine-free SEI layers can be built directly on Li by in situ polymerization with benign additives and evaluated in symmetric cells through lifetime and critical current density. For sulfur cathodes, heteroatom-doped or hybrid MXenes can be used to couple selective polysulfide adsorption with catalytic conversion, and their effectiveness should be quantified by operando measurements of shuttle and conversion and by full cells with practical sulfur loadings [41,161]. For manufacturability, prioritize roll-to-roll compatible steps such as slot-die or spray for cast films and spray-based layer-by-layer, inline UV curing for in situ routes, and wide-web electrospinning integrated with lamination, all supported by closed-loop solvent or salt recovery [18,41,56,162].

To make studies comparable and to de-risk scale-up, co-report at minimum room-temperature ionic conductivity, lithium-ion transference number, storage modulus, electronic conductivity, critical current density, termination fractions with fitting details, interlayer spacing statistics, humidity-controlled contact angles, and flammability metrics, together with full-cell stack conditions and scale-quality controls such as thickness uniformity, areal basis weight, porosity, yield, and cost per square meter [18,41,161,162].

### 8.3. Future Outlook and Research Directions

Green MXene–polymer composites can serve as a termination-programmable platform for Li-metal and Li–S cells. The same design principles can extend to Na-ion, K-ion, and Zn-ion batteries and supercapacitors, with an emphasis on safety and sustainability. However, these opportunities should be weighed against several unresolved practical issues. Many green MXenes still oxidize during storage or processing, the cost of producing large, defect-controlled flakes is high, and it remains difficult to achieve uniform surface terminations over large-area electrodes. In the near term, research should standardize fluoride-free synthesis so that -O, -OH, -Cl, and -S terminations are stable, reproducible, and well characterized, then couple these surfaces to five scalable fabrication routes, namely solution blending and film casting, in situ polymerization, surface grafting, layer-by-layer assembly and electrospinning, while preserving electronic insulation below percolation. Architectural control that organizes ion corridors at fixed thickness, for example, gradient or Janus membranes with gently pillared galleries, deserves priority because it raises conductivity without sacrificing modulus.

On the anode side, fluorine-free SEI formed by in situ polymerization on lithium should be validated under stepwise current and realistic stack pressure; on the sulfur side, bifunctional MXene motifs that combine selective adsorption with catalytic conversion need operando evidence at practical sulfur loadings; in aqueous zinc systems, hydrophilic terminations and regulation of water activity should be used to suppress gas evolution and resist dendrites. Translation to devices should rely on roll-to-roll slot-die or spray coating, spray-based layer-by-layer assembly, inline ultraviolet curing, and wide-web electrospinning integrated with closed-loop recovery of salts and solvents. These steps should be supported by inline metrology for thickness, basis weight, porosity, uniformity, through-plane conductivity, and cost per area. Benchmarking should report transport, mechanics, interphase quality, and fire safety under clearly stated electrolyte-to-sulfur ratios, negative-to-positive capacity ratios, pressure, temperature, and loading. Reporting these conditions explicitly makes results comparable and ready for scale-up. A longer-term goal is a public data library that links synthesis and termination to polymer descriptors and properties. This database, supported by model-guided design and life-cycle assessments, would enable a disciplined path from coin cells to auditable pouch-level prototypes across multiple storage technologies.

## Figures and Tables

**Table 1:** MXene synthesis routes: methods, typical etching windows, key features, pros/cons, and surface terminations.

Method	Typical Etching Window (Concentration, T, Time)	Key Features	Advantages	Disadvantages	Surface Terminations	Refs
Direct HF	20–50 wt% HF, 25 °C, 12–48 h	Concentrated HF at room temperature removes Al fast and delaminates after DMSO or TBAOH, yielding F-bearing Ti_3_C_2_T_x_.	Very fast and simple with a large know-how base.	Uses hazardous HF, gives F-rich surfaces, and risks AlF_3_·3H_2_O.	Mostly F with −OH/−O mixed.	[10,65,66]
In situ HF	6–9 M HCl with LiF, 25–40 °C, 12–48 h	LiF with HCl generates HF while Li^+^ and water pre-intercalate, giving clay-like MXene that delaminates easily and remains F-bearing.	Easier handling than neat HF and delaminates easily at scale.	Still fluorine-bearing and needs careful washing to avoid AlF_3_·3H_2_O.	−F/−OH/−O mixed, usually less F than neat HF.	[19,21,67]
Bifluoride etching	Concentrated bifluoride solution (NH_4_HF_2_, KHF_2_), 25–60 °C, 24–72 h	Bifluoride salts buffer HF at 25–60 °C and expand the galleries, providing controllable etching that is still F-bearing.	Buffered HF broadens the window and often increases interlayer spacing.	Etching is slower and fluorine remains to manage.	−F/−OH/−O mixed from buffered HF.	[23,24]
Alkali hydrothermal etching	27–30 M NaOH (or KOH), 180–280 °C, 12–48 h (autoclave)	Concentrated NaOH under hydrothermal conditions leaches Al and creates fluoride-free O or OH terminated MXene.	Fluoride-free with −O/−OH-rich, hydrophilic surfaces suited to water processing.	Requires strict control to avoid titanates and safe handling of hydrogen.	Predominantly −O/−OH, fluoride-free.	[25,29,30,31]
Acidic hydrothermal	6–12 M acid (HCl), 160–200 °C, 12–48 h (autoclave)	HCl in an autoclave complexes the A layer and yields fluoride-free MXene with mixed Cl and O or OH terminations.	Fluoride-free with tunable Cl and O terminations using simple reagents.	Demands corrosion-proof hardware and thorough chloride removal.	Mixed −Cl with −O/−OH.	[32,33]
Salt-assisted alkali etching	Concentrated NaOH with salt or mild oxidant, 270 °C, 12 h (autoclave)	NaOH with a mild oxidant accelerates leaching and forms a thin TiO_2_ spacing layer, producing fluoride-free O or OH surfaces.	Faster kinetics and a thin TiO_2_ skin that reduces restacking.	Over-oxidation can occur and chloride must be rinsed well.	−O/−OH with a thin TiO_2_ interfacial layer.	[34]
Microwave–hydrothermal	Concentrated NaOH, 160–220 °C, 30–45 min (microwave-assisted autoclave)	Microwave heating speeds alkaline hydrothermal etching near 180 °C and narrows thickness while staying fluoride-free.	Short dwell time and narrow thickness distribution without fluorides.	Needs microwave-rated reactors with uniform fields and pressure control.	−O/−OH from alkaline media.	[35,36]
Electrochemical etching	1 M NH_4_Cl + 0.2 M TMAOH, ~5 V, room temperature, ~5 h (electrochemical cell)	Chloride electrolytes under applied bias remove Al and hydroxide writes O or OH terminations with widened galleries, fluoride-free.	Fluoride-free with tunable terminations and large few-layer flakes.	Over-etching can form a CDC skin and scale-up is engineering heavy.	−O/−OH with optional −Cl depending on electrolyte.	[37,38,39]
Lewis-acid molten-salt	Lewis-acidic molten salts (ZnCl_2_/CuCl_2_), 550–800 °C, ~1.5–3 h (sealed ampoule)	Molten Lewis salts at high temperature replace Al and halogenate the surface, giving halogen-terminated MXene without HF.	HF-free with programmable Cl, Br, or I terminations and high conductivity.	Runs at high temperature and can cause melt corrosion and residues.	Designed −Cl/−Br/−I halogen terminations.	[26,44,45]
Low-temperature hydrated molten-salt	LiCl/MgCl_2_·6H_2_O (molten salt hydrate), 150 °C, ≈10–12 h (muffle furnace in air)	LiCl and MgCl_2_·6H_2_O near 150 °C in air create a semi-molten shield that etches gently and delaminates spontaneously with mixed Cl and O or OH terminations.	Mild, air-operable conditions with spontaneous delamination and polymer-friendly surfaces.	The window is narrow, yields are moderate, and an acid cleanup is needed.	Mixed −Cl and −O/−OH.	[49]
Iodine-assisted non-aqueous etching	I_2_ in anhydrous CH_3_CN (Ti_3_AlC_2_:I_2_ ≈ 1:3), 100 °C, ~4 days (sealed, halogen-assisted etching in organic solvent)	I_2_ in dry solvent forms an I-terminated intermediate that converts to −O or −OH during work-up and remains fluoride-free.	Fluoride-free route that gives oxygen-rich, stable, and conductive films.	Requires dry handling and a post-exchange and delamination step.	I-terminated intermediate that converts to −O/−OH after work-up.	[51]
Photo-Fenton soft-chemistry etching	Aqueous Na_2_C_2_O_4_/Fe^3+^ photo-Fenton solution (pH = 3, Na_2_C_2_O_4_:Fe^3+^ = 3:1) with added H_2_O_2_, room temperature, ~10 h under UV–vis irradiation (batch reactor).	Light-driven Fe and H_2_O_2_ generate radicals that remove Al under mild acidity and produce fluoride-free O or OH terminations.	Green, low-temperature chemistry that yields O/OH-rich MXene.	Residual iron and oxidants must be removed and TiO_2_ growth must be limited.	Predominantly −O/−OH, fluoride-free.	[55]
Chemical vapor deposition	Metal and halide precursors (e.g., Ti/TiCl_4_, TiCl_3_ or Mo/CH_4_), 650–1100 °C, 0.5–3 h (gas-phase CVD in quartz tube furnace)	Gas-phase growth yields Ti_2_CCl_2_ or Ti_2_NCl_2_ films with halogen terminations at wafer scale without HF.	Wafer-scale films with very low sheet resistance and precise control.	The thermal budget is high and transfer or activation is required.	As-grown −Cl on Ti_2_CCl_2_/Ti_2_NCl_2_.	[57,58,59]
Mechanochemical	0.25 M LiCl + 1 M TMAOH aqueous etchant, ball-milled with Ti_3_AlC_2_ at 400 rpm (15 min reverse rotation), room temperature (chemical-combined ball-milling, fluorine-free porous Ti_3_C_2_).	Near-dry ball-milling with base and salt couples −OH etching with defect-assisted delamination and gives fluoride-free O or OH terminations.	Solvent-lean and scalable with high-area −O/−OH- rich flakes.	Contamination and oxidation can occur and yields and terminations vary.	Predominantly −O/−OH with defect-rich edges.	[62,64]

**Table 2:** MXene reporting checklist: surface chemistry, ion transport, interfacial reactivity, mech/thermal, process controls.

Pillars	Must Report	Measurements	Link to Device Metrics	Practical and Green Controls
Terminations and wetting	Termination ensemble by XPS, surface energy and contact angles, solvent system noted as NMP-free or not	XPS with fitting notes, advancing and receding angles at controlled humidity, zeta potential for dispersion stability	Interfacial impedance for Li-facing layers, ESR for Li–S electrodes, electrolyte uptake and wetting time	Limit oxidation during dispersion, shorten water dwell time, use antioxidants when compatible, match O- or OH-rich MXene to ether or nitrile matrices, match Cl- and O-rich MXene to fluoropolymers or ionogels
2D ion pathways and spacing	d(002) and its humidity dependence, flake size and thickness distributions, alignment or tortuosity indicator	XRD or SAXS with humidity control, temperature-dependent impedance for activation energy, microscopy for alignment and porosity	Ionic conductivity at stated thickness, CCD and Li^+^ flux uniformity in LMB, rate retention at matched thickness in Li–S	Remove interlayer water after casting, use gentle intercalants or pillaring to prevent restacking, pair green MXene with short aqueous steps, use low-polarity binders for molten-salt MXene
Interfacial reactivity and interphase control	Nucleation overpotential, interfacial resistance evolution, CE protocol, post-cycling chemistry and morphology	Galvanostatic CE tests with defined current and areal capacity, EIS before and after cycling, XPS or ToF-SIMS and SEM or TEM	CCD and overpotential in LMB, shuttle suppression and areal utilization in Li–S, stable impedance under rate changes	Match terminations to polymer and salt, prefer fluoride-free routes when performance is comparable so LiF arises from controlled salt breakdown, document electrolyte and binder to separate route effects from formulation
Mechanics and heat management	Storage modulus or tensile metrics on the same films, in-plane or through-film thermal conductivity, electronic conductivity at intended loading	DMA or tensile testing, laser-flash or steady-state thermal conductivity, four-probe conductivity	Shape stability and resistance to filament penetration in LMB, temperature rise at power in Li–S and LMB, long-cycle retention at matched power	Keep electronic networks below percolation in electrolyte-rich regions, use aqueous or alcohol processing for green MXene to preserve aspect ratio, consider PVDF latex to avoid NMP, align or grade platelets to boost modulus and heat spreading at low loading
Cross-cutting moderators	Electronic percolation restraint, preservation of surface chemistry	Four-probe conductivity at loading of interest, oxidation and humidity indicators over storage time	Self-discharge and parameter drift with storage and humidity	Inert storage, humidity control, short wet-processing steps, log time from synthesis to casting

**Table 3:** MXene–polymer fabrication methods: process, interfaces, morphology, mechanics, scalability, and pitfalls.

Method	Process Type	Interface Chemistry	Morphology Control	Mechanical Compatibility	Scalability	Common Pitfalls
Solution blending and casting	Wet mixing then cast or coat	Mainly physical interactions, hydrogen bonding, van der Waals	Film or coating thickness tuned by solids and shear, some flake alignment by coating	Moderate, depends on dispersion and loading	High roll to roll slot, die blade or spray coating straightforward, solvent recovery needed	Restacking, brittle film at high loading, uneven thickness
In situ polymerization	Polymerization in presence of MXene thermal or UV	Stronger interfacial contact chains form near flakes, possible covalent links	Dense networks around flakes, good embedment	High, good load transfer and cohesion	Moderate batch or inline curing, feasible chemistry and oxygen sensitivity can limit throughput	MXene oxidation, residual monomer, oxygen sensitivity, shrinkage
Surface grafting-to or -from	Covalent functionalization and chain attachment	Covalent brushes or tethers on MXene	Brush layers prevent restacking, interface tailored	Excellent, very strong adhesion	Low to moderate multistep wet chemistry, washing and control of graft density, slow for large area	Multistep complexity, low throughput, over-grafting reduces conductivity
Layer-by-layer assembly	Alternating deposition of oppositely charged species	Electrostatic hydrogen bonding, secondary interactions	Nanometer-level thickness, highly ordered laminar stacks	Good but can be brittle through-thickness	Low cyclic dipping or spray LbL is slow, automation helps but still limited	Slow cycles, rinse defects, substrate dependence
Electrospinning	High voltage fiber formation from polymer MXene dope	Physical embedment, affinity driven	3D porous nonwoven fiber alignment via collector	High, flexible and tough in plane	Moderate multi-needle or needleless setups give m^2^ scale solvent handling and safety required	Jet clogging, solvent hazards, thickness control, MXene aggregation

## Data Availability

No new data were created or analyzed in this study.
